# Gut microbiota in health and disease: advances and future prospects

**DOI:** 10.1002/mco2.70012

**Published:** 2024-11-20

**Authors:** Yusheng Zhang, Hong Wang, Yiwei Sang, Mei Liu, Qing Wang, Hongjun Yang, Xianyu Li

**Affiliations:** ^1^ Beijing Key Laboratory of Traditional Chinese Medicine Basic Research on Prevention and Treatment for Major Diseases Experimental Research Center China Academy of Chinese Medical Sciences Beijing China; ^2^ School of Traditional Chinese Medicine Southern Medical University Guangzhou China; ^3^ School of Life Sciences Beijing University of Chinese Medicine Beijing China; ^4^ State Key Laboratory for Quality Ensurance and Sustainable Use of Dao‐di Herbs China Academy of Chinese Medical Sciences Beijing China

**Keywords:** cell death, ferroptosis, gut microbiota, microbiome–gut–organ axis chip, pyroptosis, single‐bacterium omics

## Abstract

The gut microbiota plays a critical role in maintaining human health, influencing a wide range of physiological processes, including immune regulation, metabolism, and neurological function. Recent studies have shown that imbalances in gut microbiota composition can contribute to the onset and progression of various diseases, such as metabolic disorders (e.g., obesity and diabetes) and neurodegenerative conditions (e.g., Alzheimer's and Parkinson's). These conditions are often accompanied by chronic inflammation and dysregulated immune responses, which are closely linked to specific forms of cell death, including pyroptosis and ferroptosis. Pathogenic bacteria in the gut can trigger these cell death pathways through toxin release, while probiotics have been found to mitigate these effects by modulating immune responses. Despite these insights, the precise mechanisms through which the gut microbiota influences these diseases remain insufficiently understood. This review consolidates recent findings on the impact of gut microbiota in these immune‐mediated and inflammation‐associated conditions. It also identifies gaps in current research and explores the potential of advanced technologies, such as organ‐on‐chip models and the microbiome–gut–organ axis, for deepening our understanding. Emerging tools, including single‐bacterium omics and spatial metabolomics, are discussed for their promise in elucidating the microbiota's role in disease development.

## Graphical abstract

This review discusses how gut microbiota influences metabolic and neurological diseases, then delves into the specific immune inflammation mechanisms related to cell death, such as pyroptosis and ferroptosis. Advanced technologies like organ‐on‐chip, single‐bacterium transcriptomics, and spatial metabolomics are highlighted for their potential in exploring these mechanisms.

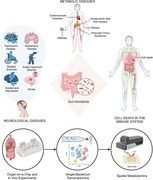

## 1 INTRODUCTION

The gut microbiota mainly consists of Firmicutes, Bacteroidetes, Actinobacteria, and Proteobacteria, with Firmicutes and Bacteroidetes comprising about 90%. These microbes play a crucial role in food digestion, metabolism, maintaining the intestinal barrier, and regulating the immune system. The gut contains approximately 100 trillion (10^14^) microorganisms, and their gene pool is larger than the human genome. Increasing evidence suggests a bidirectional interaction between human genes and microbial genes. A healthy gut microbiota is typically diverse and functionally stable, but defining a “healthy” microbiota is complicated by differences in individual physiological states.^1^, ^2^ The gut microbiota is closely linked to the onset and progression of various diseases. Research increasingly shows that gut microbes not only play a crucial role in maintaining host health but also contribute to the pathological processes of complex human diseases, including metabolic disorders, neurodegenerative diseases, immune‐inflammatory diseases, and cancer. For instance, gut dysbiosis can trigger inflammation and metabolic dysfunction, which are strongly associated with obesity, diabetes, inflammatory bowel disease (IBD), and neurodegenerative disorders.^3^, ^4^ The gut microbiota influences disease progression by regulating the host's immune response and metabolism, either directly or indirectly.^5^ Therefore, changes in the composition of gut microbes and their metabolic products have potential applications in the diagnosis and treatment of diseases.

Cell death is crucial for maintaining tissue homeostasis during normal development and for mounting effective responses to pathogens. Apoptosis, pyroptosis, and ferroptosis are the most extensively studied forms of regulated cell death, although new forms continue to be identified.^6^ The relationship between the gut microbiota and cell death is complex. The gut microbiota influences cell death by regulating the host immune response, maintaining intestinal barrier function, and resisting pathogen invasion. A healthy gut microbiota can suppress excessive inflammation and prevent excessive cell death, thereby preserving tissue integrity. However, when the gut microbiota becomes dysregulated, the overgrowth of harmful microbes may trigger excessive cell death, leading to inflammation and the development of related diseases.^7^ Studies have shown that when intestinal epithelial cells (IECs) begin to die, they release certain nutrients that can be sensed and utilized by gut microbes, such as *Salmonella* and *Escherichia coli*. This phenomenon, known as “Death‐Induced Nutrient Release,” occurs in various disease contexts. Gut bacteria exploit these nutrients to aid their colonization, potentially leading to issues such as food poisoning, inflammatory diseases, and chemotherapy‐induced mucositis.^8^ Additionally, the gut microbiota can influence cell death pathways, such as pyroptosis and ferroptosis, through its metabolic products. This plays a crucial role in the development of inflammatory diseases and tumors.^9^, ^10^

However, there is relatively little literature specifically focusing on the mechanisms linking gut microbiota and necrosis‐related diseases. Most studies focus on intestinal diseases such as colorectal cancer (CRC) and IBD. Although some studies explore correlations with distal organs, their findings tend to lack depth and are less convincing. This can be attributed to the complexity of the gut–organ axis, which involves highly dynamic and multidimensional interactions between the gut and other organs. Gut microbiota influence distant organs such as the brain, liver, and lungs through microbial metabolites. Studying these axes requires the integration of disciplines like microbiology, immunology, and neuroscience, which increases the complexity of both study design and data analysis. Additionally, the gut–organ axis involves multiple signaling pathways, including metabolic, endocrine, immune, and neural pathways, whose cross‐regulation further complicates research.^11^, ^12^

Therefore, this review not only elucidates the relationship between gut microbiota and metabolic and neurological diseases but also summarizes recent literature on cell death‐related diseases closely associated with immune system inflammation and the gut microbiota. Furthermore, it highlights how advanced integrated technologies offer comprehensive approaches to understanding the influence of gut microbiota on cell death. The review emphasizes the significance of organ‐on‐a‐chip technology, spatial omics, and single‐bacterium transcriptomics in studying the impact of gut microbiota on cell death.

## 2 GUT MICROBIOTA AND METABOLIC DISEASES

The gut microbiota plays a crucial role in the development and progression of various metabolic diseases, including obesity, type 2 diabetes (T2DM), nonalcoholic fatty liver disease (NAFLD), and polycystic ovary syndrome (PCOS). Studies have shown significant alterations in the gut microbiota composition in obese individuals, with decreased levels of *Akkermansia*, *Faecalibacterium*, *Oscillibacter*, and *Alistipes*, while obesity‐associated microbiota have an enhanced ability to extract energy from the diet. Metabolites produced by the gut microbiota, such as bile acids (BAs) and short‐chain fatty acids (SCFAs), are closely linked to host metabolism. SCFAs like acetate, propionate, and butyrate regulate energy and glucose metabolism, as well as appetite, through interactions with G‐protein‐coupled receptors. In obese patients, reduced microbial diversity and diminished SCFA‐producing bacteria exacerbate metabolic disturbances.^13^, ^14^

In T2DM patients, a reduction in butyrate‐producing bacteria and an increase in proinflammatory species are observed, with certain metabolites like indolepropionic acid being associated with improved insulin sensitivity. The gut microbiota also influences insulin resistance by modulating SCFA production and intestinal permeability. Cardiometabolic diseases are linked to elevated levels of gut microbiota‐derived trimethylamine N‐oxide (TMAO), which promotes atherosclerosis (AS) and is closely associated with an increased risk of cardiovascular diseases (CVDs). In NAFLD patients, there is a notable shift in the gut microbiota, with an increase in *Bacteroidetes* and a decrease in *Firmicutes* (particularly SCFA‐producing species), along with reduced microbial diversity that correlates with disease severity.^15^, ^16^, ^17^

Similarly, PCOS patients exhibit altered gut microbiota composition, with decreased abundances of *Lactobacillus*, *Ruminococcus*, and *Clostridium*, and increased levels of *Prevotella*, *Bacteroides*, *Escherichia/Shigella*, and *Streptococcus*. BAs, as key metabolic regulators, interact with the gut microbiota to influence systemic metabolism by activating signaling pathways like the farnesoid X receptor (FXR), thus regulating lipid metabolism.^18^, ^19^

Metabolic syndrome, characterized by abnormalities such as CVD, obesity, and insulin resistance, is closely associated with gut microbiota dysbiosis, including an altered ratio of *Firmicutes* to *Bacteroidetes*. Metabolites such as SCFAs and TMAO produced by the gut microbiota directly impact the host's energy balance and metabolic health. Elevated TMAO levels have been linked to CVDs and metabolic syndrome. Moreover, the gut microbiota's metabolism of dietary choline and carnitine, leading to increased TMAO levels, is closely associated with obesity and insulin resistance.^20^, ^21^

Gut dysbiosis can also contribute to the progression of metabolic liver diseases, such as NAFLD, through mechanisms involving lipid metabolism and BA synthesis. The imbalance in gut microbiota composition and function, particularly the production of BAs and SCFAs, is a critical factor in NAFLD development. Restoring gut microbiota balance, through dietary interventions, probiotics, or fecal microbiota transplantation, has the potential to improve metabolic health and prevent the onset of metabolic diseases.^22^

The literature have explored the impact of clinical drugs used to treat metabolic diseases on gut microbiota, revealing notable effects on microbial composition. Key drugs, such as metformin, statins, and proton pump inhibitors, have been shown to induce significant shifts in gut bacterial populations. Metformin, a widely prescribed drug for T2DM, has been linked to an increased abundance of *Akkermansia muciniphila* and SCFAs‐producing bacteria like *Lactobacillus*. These changes are believed to contribute to its beneficial effects on glucose metabolism and overall metabolic health. However, metformin's impact on the gut microbiota may also lead to gastrointestinal side effects, as the altered microbial balance affects intestinal function. Statins, which are commonly used to lower cholesterol, have been found to increase the relative abundance of *Bacteroidetes* and decrease *Firmicutes*. This shift is associated with improved lipid metabolism and reduced systemic inflammation, contributing to enhanced cardiovascular outcomes. However, in some patients, this alteration in microbial composition may lead to dysbiosis, potentially causing metabolic complications. Similarly, PPIs, frequently prescribed for acid reflux, have been shown to significantly reduce gut microbial diversity. This reduction is accompanied by an increase in potentially harmful bacteria, such as *Enterococcus*, *Streptococcus*, and *E. coli*, which can elevate the risk of gastrointestinal infections.^23^, ^24^, ^25^, ^26^

## 3 GUT MICROBIOTA AND NEUROLOGICAL DISEASES

The relationship between gut microbiota and neurological diseases has become a major focus of research. A growing body of evidence shows that there is complex, bidirectional communication between the gut microbiota and the central nervous system (CNS), known as the gut–brain axis (GBA). Gut microbiota regulates host metabolism, immune function, and neural processes by producing metabolites like SCFAs, BAs, amino acids, and neurotransmitters, maintaining a healthy physiological state. However, when the gut microbiota becomes dysregulated, it can exacerbate the progression of neurological diseases through inflammation and metabolic disorders.^27^

The gut microbiota affects brain function through neural, immune, endocrine, and metabolic pathways. Through the neural route, the gut communicates directly with the CNS via the vagus nerve, and microbiota‐produced neurotransmitters (such as gamma‐aminobutyric acid (GABA), serotonin, and acetylcholine) modulate brain activity. The immune pathway involves SCFAs and cytokines produced by the gut microbiota, which influence neuroinflammation and cross the blood–brain barrier (BBB) to affect CNS function. The endocrine pathway includes hormones such as GLP‐1 and PYY, which regulate brain functions related to memory, mood, and cognition. Metabolic pathways involve SCFAs, BAs, and other metabolites that directly or indirectly impact neurotransmitter levels, regulating neural signaling and behavior.^28^, ^29^

In Alzheimer's disease (AD), gut dysbiosis is characterized by increased *Bacteroidetes* and decreased *Firmicutes* (like *Ruminococcaceae* and *Clostridiaceae*). These changes are linked to increased inflammatory markers (e.g., IL‐1β and IL‐18), which exacerbate AD pathology. Lower SCFA levels in the gut also contribute to neuroinflammation, suggesting a direct role for gut microbiota in the disease's progression. Additionally, lower levels of SCFAs, produced by beneficial gut bacteria, have been linked to increased neuroinflammation and amyloid‐β (Aβ) plaque formation, further accelerating AD development. Dysbiosis also leads to increased gut permeability, allowing harmful substances like lipopolysaccharides (LPS) to enter circulation, cross the BBB, and trigger neuroinflammatory responses.^30^, ^31^

Similarly, in Parkinson's disease (PD), gut microbiota diversity is reduced, particularly SCFA‐producing bacteria like *Lachnospiraceae* and *Faecalibacterium prausnitzii*. *Akkermansia* is increased, leading to heightened gut permeability and the leakage of toxins such as LPS into the CNS, promoting α‐synuclein aggregation. Gut dysbiosis may also directly communicate with the brain via the vagus nerve or through inflammatory pathways, worsening PD pathology.^32^, ^33^

Multiple sclerosis is a neuroinflammatory disorder marked by demyelination. Multiple sclerosis patients exhibit altered gut microbiota, with an increase in *Firmicutes* and a reduction in *Fusobacteria*. A decrease in SCFA‐producing bacteria has also been linked to exacerbated inflammation, highlighting the role of the gut microbiota in driving neuroinflammation and demyelination. Additionally, gut microbiota‐derived molecules are implicated in modulating both innate and adaptive immunity, particularly in the regulation of T cells. These cells play a crucial role in multiple sclerosis, where excessive inflammation leads to damage in the CNS. Therapies targeting gut microbiota, such as probiotics or personalized nutrition, are being explored as potential strategies to mitigate the neuroinflammatory processes involved in multiple sclerosis.^34^, ^35^, ^36^

In autism spectrum disorder (ASD), gut dysbiosis alters SCFA levels and neurotransmitter metabolism, affecting pathways such as GABA, glutamate (Glu), and serotonin. ASD patients tend to have lower gut microbial diversity, with reductions in *Firmicutes* and *Clostridiales* and increased *Prevotella* and *Bifidobacterium*, which may contribute to ASD pathology. Similarly, anxiety and depression are associated with gut microbiota changes, including reductions in SCFA‐producing bacteria like *Faecalibacterium* and *Eubacterium rectale*, and increases in proinflammatory strains such as *Ruminococcus* and *Escherichia/Shigella*. Probiotic supplementation has been shown to alleviate depressive symptoms, underscoring the microbiota's role in mood regulation.^37^, ^38^

In stroke patients, gut microbiota changes are also significant, with decreases in *Faecalibacterium* and *Prevotella*, and increases in opportunistic pathogens like *Desulfovibrio* and *Enterobacter*. SCFA metabolites may reduce neuroinflammation and promote neural repair, affecting post‐stroke recovery.^39^, ^40^

Additionally, antidepressants and antipsychotics have been shown to influence gut microbiota. For instance, selective serotonin reuptake inhibitors increase the abundance of specific bacteria like *Eubacterium ramulus*, while tricyclic antidepressants are associated with increased *Clostridium leptum*. These changes in gut microbiota may enhance the therapeutic effects of these drugs by promoting SCFA production, which has anti‐inflammatory properties and supports gut barrier function. Antipsychotics such as risperidone and olanzapine have also been linked to significant changes in gut microbiota composition, with *Firmicutes* increasing and *Bacteroidetes* decreasing, changes that are associated with metabolic disturbances like weight gain.^41^

## 4 CELL DEATH

### 4.1 Apoptosis

Apoptosis has long been regarded as a nonlytic and immunologically silent form of cell death, aimed at maintaining tissue health by eliminating unnecessary or damaged cells. It can be initiated through intrinsic or extrinsic pathways. The intrinsic pathway is usually triggered by intracellular stress or damage signals, such as DNA damage or the absence of growth factors, with the B‐cell lymphoma 2 (Bcl‐2) family of proteins playing a key role in balancing proapoptotic and antiapoptotic signals. Activation of proapoptotic factors increases mitochondrial outer membrane permeability, releasing cytochrome *c*, which then activates caspase proteases to execute cell death. The extrinsic pathway is initiated by external death signals, such as tumor necrosis factor (TNF) or Fas ligand, binding to specific receptors, which activates initiator caspases like caspase‐8, subsequently activating downstream executioner caspases like caspase‐3, leading to cellular degradation and DNA fragmentation. Caspase‐3 and caspase‐7, as executioner proteases, are responsible for cleaving various intracellular substrates, leading to morphological changes such as cell shrinkage, chromatin fragmentation, membrane blebbing, and the formation of apoptotic bodies. They also cleave DNA fragmentation factor, promoting the cleavage of chromatin DNA into nucleosomal fragments. Additionally, caspase‐3 cleaves phospholipid flippases such as Xk‐related protein 8 (XKR8), resulting in the exposure of phosphatidylserine on the outer membrane, allowing noninflammatory phagocytic recognition. Second mitochondria‐derived activator of caspases/Direct IAP‐binding protein with low pI (Smac/Diablo) is released from mitochondria to relieve the inhibition of caspases by inhibitor of apoptosis proteins (IAP) proteins, further driving apoptosis. BH3 interacting‐domain death agonist (BID) is cleaved by caspase‐8 into truncated BID (tBID), which triggers mitochondrial outer membrane permeability, releasing apoptotic proteins. Moreover, receptor‐interacting protein kinase 1 (RIPK1) not only functions as an adaptor protein in apoptosis but also plays a role in necroptosis by activating receptor‐interacting protein kinase 3 (RIPK3), demonstrating its dual role in regulating cell death.^42^, ^43^

### 4.2 Ferroptosis

**FIGURE 1:**
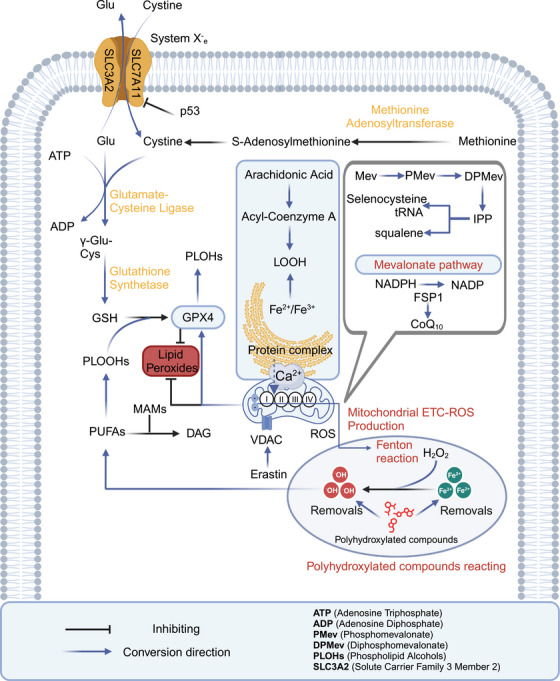
Mechanisms regulating ferroptosis: pathways and cellular interaction. Ferroptosis is a form of cell death linked to iron ions and characterized by PUFA peroxidation and ROS increase. The Cys/Glu–GSH–GPX4 pathway is crucial for inhibiting ferroptosis. In this pathway, GSH synthesis from methionine maintains GPX4 activity, which neutralizes LOOHs that execute ferroptosis. Ferroptosis inducers like Erastin cause mitochondrial dysfunction via VDACs, while p53 downregulates SLC7A11, affecting GPX4 and reducing cellular antioxidant capacity. Polyhydroxy compounds, when combined with iron ions, form free radicals, promoting harmful lipid peroxidation. GSH depletion weakens GPX4, increasing susceptibility to ferroptosis. The Mev pathway contributes to ferroptosis by producing IPP, squalene, CoQ10, and cholesterol, which are linked to GPX4 function and antioxidant potential. Phosphatidylethanolamine rich in PUFAs serves as a key substrate for ferroptosis, with ACSL4 and LPCAT3 enzymes playing a role in PUFA incorporation into membrane lipids. Mitochondria–ER interaction, particularly through MAMs, regulates ferroptosis by controlling calcium ion flux and lipid remodeling.

Ferroptosis and pyroptosis are caused by lipid peroxidation and the formation of pores by gasdermins (GSDMs) or mixed lineage kinase domain‐like pseudokinase. These processes lead to cell membrane rupture and trigger a strong inflammatory response. Specifically, ferroptosis is a form of cell death driven by the accumulation of iron‐dependent lipid peroxides (LOOHs), such as malondialdehyde (MDA) and 4‐hydroxy‐2‐nonenal (4‐HNE). Polyunsaturated fatty acids (PUFAs) in membrane phospholipids promote ferroptosis through lipid peroxidation, while monounsaturated fatty acids (MUFAs) inhibit this process by reducing reactive oxygen species (ROS) production. The cystine (Cys)/Glu antiporter (system Xc^−^)–glutathione (GSH)–glutathione peroxidase 4 (GPX4) pathway plays a key role in resisting ferroptosis. In this pathway, GSH synthesis from methionine maintains GPX4 activity, which neutralizes LOOHs that execute ferroptosis. LOOH is generated from lipid hydroperoxidation and plays a central role in ferroptosis progression. Ferroptosis inducers like erastin target the voltage‐dependent anion channel (VDAC), causing mitochondrial dysfunction and the release of oxidative substances, which lead to oxidative cell death. Additionally, tumor protein p53 (p53) triggers ferroptosis by inhibiting solute carrier family 7 member 11 (SLC7A11) expression, reducing Cys uptake, lowering GSH peroxidase activity, and increasing lipid ROS. SLC7A11 is a key component of the system Xc^−^, located on the cell membrane, primarily responsible for importing extracellular Cys into the cell while exporting intracellular Glu. The nonenzymatic, iron‐dependent Fenton reaction is also a key mechanism in ferroptosis. When GPX4 activity is inhibited, phospholipid hydroperoxides (PLOOH) accumulate, accelerating the Fenton reaction and heightening susceptibility to ferroptosis. The diacylglycerol pathway facilitates the transport of PUFA‐derived lipids, feeding into the LOOH formation process depicted. Polyhydroxy compounds, when combined with iron ions, form free radicals, promoting harmful lipid peroxidation. GSH depletion weakens GPX4, increasing susceptibility to ferroptosis. The mevalonate (Mev) pathway contributes to ferroptosis by producing isopentenyl pyrophosphate (IPP), squalene, squalene, coenzyme Q10 (CoQ10), and cholesterol, which are linked to GPX4 function and antioxidant potential. The ferroptosis suppressor protein 1 (FSP1)–nicotinamide adenine dinucleotide phosphate hydrogen (NADPH)–CoQ10 pathway also plays a role in inhibiting ferroptosis by regenerating CoQ10 and suppressing lipid peroxidation. FSP1 catalyzes the reduction of CoQ10 using NADPH, which then functions as a lipid‐soluble antioxidant to prevent the buildup of PLOOHs. The generation of PLOOHs is central to ferroptosis, and CoQ10 plays a key role in neutralizing these LOOHs. Phosphatidylethanolamin rich in PUFAs serves as a key substrate for ferroptosis, with Acyl‐CoA synthetase long chain family member 4 (ACSL4) and lysophosphatidylcholine acyltransferase 3 (LPCAT3) enzymes playing a role in PUFA incorporation into membrane lipids. Mitochondria–endoplasmic reticulum (ER) interaction, particularly through mitochondria‐associated membrane (MAM), regulates ferroptosis by controlling calcium ion flux and lipid remodeling (Figure 1).^44^, ^45^, ^46^, ^47^, ^48^, ^49^, ^50^

### 4.3 Pyroptosis

**FIGURE 2:**
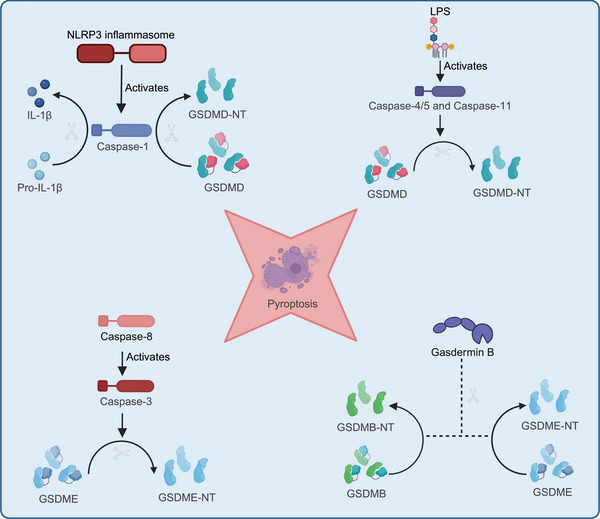
Pyroptosis pathways: four distinct forms. Pyroptosis occurs through four distinct molecular pathways, all centered on the activation of GSDM proteins, which ultimately lead to pore formation in the cell membrane and inflammatory cell death. In the canonical pathway, the NLRP3 inflammasome activates caspase‐1, which cleaves GSDMD into its GSDMD‐NT, forming membrane pores and releasing IL‐1β to propagate inflammation. In the noncanonical pathway, LPS directly activate caspase‐4/5 (in humans) or caspase‐11 (in mice), which also cleave GSDMD, driving pyroptosis. Another form of pyroptosis involves caspase‐8, which activates caspase‐3, leading to the cleavage ofGSDME, allowing its N‐terminal fragment to form pores and induce cell death. Finally, GSDMB can also be cleaved by caspases, including caspase‐3, leading to pyroptotic cell death via GSDMB‐NT pore formation.

Pyroptosis is mediated by inflammasomes, such as NOD‐like receptor protein 3 (NLRP3), absent in melanoma 2 (AIM2), and NOD‐like receptor family CARD domain containing 4 (NLRC4), which ultimately activate caspase‐1 or caspase‐11, leading to a series of cellular changes. During infection or stress, inflammasomes like NLRP3 are activated by detecting intracellular and extracellular danger signals, such as pathogens, toxins, or ATP. These signals can be exogenous, like bacterial LPS, or endogenous, such as cellular damage signals. The activation of inflammasomes leads to the activation of caspase‐1 or caspase‐11. Caspase‐1 is a key effector protease that cleaves and activates precursors of inflammatory cytokines, such as pro‐IL‐1β and pro‐IL‐18, to produce their mature forms, IL‐1β and IL‐18. Caspase‐1 also cleaves GSDM D (GSDMD), releasing its N‐terminal fragment, which creates pores in the cell membrane, disrupting membrane integrity. This pore formation causes water and ion imbalances, ultimately leading to cell lysis. As the cell lyses, large amounts of proinflammatory cytokines like IL‐1β and IL‐18 are released into the extracellular space, amplifying the local inflammatory response. In addition to the classical pyroptosis pathway, which is triggered by inflammasomes like NLRP3 activating caspase‐1 or caspase‐11, other members of the GSDM family, such as GSDME and GSDMB, can also induce pyroptosis through cleavage by caspase‐3 or granzymes. The noncanonical pyroptosis pathway is initiated by caspase‐4/5 (in humans) and caspase‐11 (in mice), which directly recognize intracellular LPS. The cleaved N‐terminal fragment of GSDMD binds to acidic phospholipids, forming pores in the cell membrane, leading to membrane rupture and the release of danger signals. Additionally, caspase‐8 or granzyme B can also induce pyroptosis by cleaving GSDM proteins, highlighting the diversity and complexity of pyroptosis in regulating cell death (Figure 2).^9^, ^43^ Lytic cell death releases damage‐associated molecular patterns (DAMPs), which strongly affect the surrounding environment and trigger a robust inflammatory response.^51^, ^52^ Dysregulated cell death is closely associated with chronic inflammation and the progression of acute diseases. In chronic inflammation, lytic cell death, such as pyroptosis, often leads to cell membrane rupture and the release of DAMPs, amplifying immune system activation. This persistent inflammatory response can drive the onset and progression of various chronic diseases, such as rheumatoid arthritis and AS. In acute conditions, such as infectious diseases, dysregulated cell death can also enable pathogen escape, worsening disease severity. Although these cell death pathways have distinct molecular mechanisms, they exhibit extensive cross‐regulation, further exacerbating the inflammatory response.^53^

## 5 RESEARCH ON GUT MICROBIOTA IN DISEASES RELATED TO CELL DEATH

IBD, which includes Crohn's disease and ulcerative colitis (UC), consists of persistent and relapsing inflammatory conditions affecting the gastrointestinal system. These disorders are characterized by damage to the intestinal mucosal barrier, leading to inicreased permeability. This permits pathogens and endotoxins to enter the intestinal lumen, provoking a strong immune response and exacerbating inflammation.^54^

Studies show that patients with IBD often experience gut dysbiosis, leading to a reduction in beneficial bacteria and SCFAs like butyrate, which increases intestinal permeability and inflammation risk. Changes in specific bacterial groups, such as reduced *Firmicutes* and *Bacteroides*, directly contribute to intestinal inflammation. Probiotics like *Saccharomyces Boulardii* and *E. coli* Nissle 1917 have demonstrated anti‐inflammatory effects in IBD treatment by enhancing intestinal barrier function and preventing pathogen adhesion.^55^, ^56^ Multistrain probiotics, such as VSL#3, have also been effective in inducing and maintaining remission in UC patients and may reduce CRC risk. The overexpression of toll‐like receptor 2 (TLR2) and TLR4 receptors in IBD patients triggers inappropriate immune responses, further exacerbating intestinal inflammation. Therefore, modulating the gut microbiota is a key therapeutic strategy for IBD.^57^

The gut microbiota plays a crucial role in the development and progression of CRC. Studies show that the gut microbiota in CRC patients differs significantly from that in healthy individuals. The abundance of specific bacteria, such as *Fusobacterium nucleatum*, *E. coli*, and *Bacteroides fragilis*, is notably increased in CRC patients. These microbes promote CRC progression by activating signaling pathways like nuclear factor kappa B (NF‐κB) and Wnt/β‐catenin, which drive inflammation, DNA damage, and tumor cell proliferation.^58^ Additionally, gut microbial metabolites play key roles in CRC. BAs, such as deoxycholic acid and lithocholic acid, are closely associated with CRC progression by inducing oxidative stress and DNA damage, promoting tumor proliferation and inflammation. Conversely, SCFAs like butyrate exhibit anticancer effects by inhibiting histone deacetylases, maintaining tight epithelial junctions, and protecting the epithelial barrier, thereby preventing tumor formation. TMAO is also linked to an increased risk of CRC by promoting inflammation and oxidative stress. Amino acid metabolism, particularly indole derivatives from tryptophan, influences CRC progression by modulating immune responses and suppressing inflammation. Gut microbiota and their metabolites hold significant potential in CRC diagnosis and treatment.^59^ *F. nucleatum* can serve as a biomarker for CRC, with its abundance closely correlated with patient prognosis. Changes in gut microbiota composition can also significantly affect the efficacy of chemotherapy and immunotherapy. Probiotics and prebiotics show promise in CRC prevention and treatment, particularly in enhancing the effectiveness of chemotherapy drugs, such as 5‐fluorouracil, and overcoming drug resistance.^60^

### 5.1 Mechanisms of pyroptosis in intestinal flora and gastrointestinal diseases

In IBD, chronic inflammation and bacterial infections are major triggers of pyroptosis. The excessive activation of the NLRP3 inflammasome in IECs and immune cells, especially macrophages, leads to an overactive pyroptotic response.^9^ The inflammasome indeed plays a central role in the apoptosis network, acting as a key pattern recognition receptor (PRR) within the immune system. It is activated when cells face stress, infection, or damage, triggering a cascade of downstream pyroptotic responses. In the gastrointestinal tract, inflammasomes are crucial not only for defending against infections but also in the development of chronic inflammation, IBD, and cancer. Notably, there is a complex bidirectional regulatory relationship between inflammasomes and the gut microbiota. Inflammasomes influence disease progression by regulating the composition of the gut microbiota and the secretion of microbial metabolites.^7^ For instance, the NLRP6 inflammasome senses microbial metabolites like taurine, driving IL‐18 and antimicrobial peptide secretion to maintain a healthy gut microbiota and antitumor immunity. Conversely, other metabolites such as histamine and putrescine can inhibit NLRP6‐dependent IL‐18 secretion, modulating inflammation and cancer progression. Additionally, SCFAs like butyrate exert anticolitis effects by activating the NLRP3 inflammasome, while diets high in cholesterol and saturated fats promote NLRP3 activation, exacerbating inflammation and tumor burden. The absence of NLRP6 is associated with an increased relative abundance of *Prevotellaceae* and *Candidatus Saccharibacteria* in the gut, which correlates with greater susceptibility to colitis, while a decrease in *Lactobacillus* spp. may heighten colitis risk. These gut microbial traits can be transmitted through cross‐fostering or cohousing, further highlighting the central regulatory role of inflammasomes in maintaining gut microbiota homeostasis and influencing disease progression.^61^

Gut dysbiosis also plays a crucial role in pyroptosis‐related intestinal diseases. In patients with IBD, dysbiosis is common, particularly an increase in proinflammatory bacteria such as enteropathogenic *E. coli*. Pyroptosis, as an inflammatory form of programmed cell death, can trigger host immune responses through the release of inflammatory mediators, clearing infected cells and restoring gut microbial balance. However, excessive activation of pyroptosis can disrupt intestinal barrier integrity, exacerbating inflammation and contributing to the progression of IBD. The relationship between specific bacteria and pyroptosis plays a pivotal role in IBD. For instance, enteropathogenic *E. coli* induces macrophage pyroptosis via an NLRP3 inflammasome‐dependent mechanism, releasing IL‐1β and triggering inflammation. Toxins produced by *E. coli*, such as Shiga toxin 2 and LPS, activate the NLRP3 inflammasome and GSDMD, inducing pyroptosis and promoting the secretion of proinflammatory cytokines.^62^ Other bacteria, such as enterotoxigenic *B. fragilis*, regulate the NLRP3 inflammasome through ROS and butyrate, promoting pyroptosis and potentially contributing to IBD, while nontoxigenic *B. fragilis* exhibits anti‐inflammatory effects by inhibiting NLRP3‐mediated inflammatory signaling pathways to reduce intestinal inflammation.^63^ *Salmonella* induces pyroptosis by recognizing LPS via TLR4 and flagellin via TLR5, activating inflammasomes such as NLRP3, NLRC4, and AIM2, leading to the production of IL‐1β and IL‐18.^64^, ^65^ This protective mechanism limits pathogen proliferation during infection. Additionally, *F. nucleatum* and *Campylobacter jejuni* also activate inflammasomes and pyroptosis pathways, impacting the progression of IBD.^66^ In summary, these bacteria and their metabolites significantly influence the progression of intestinal diseases, particularly the pathogenesis of IBD, by modulating pyroptosis. In NLRP3‐ or apoptosis‐associated speck‐like protein containing a CARD (ASC)‐deficient mice, the overgrowth of *Prevotellaceae* is associated with severe dextran sodium sulfate (DSS)‐induced colitis, likely due to decreased IL‐18 production, which compromises immune defense and epithelial integrity, worsening inflammation and disrupting pyroptosis regulation. AIM2 triggers pyroptosis by recognizing intracellular double‐stranded DNA and inhibits cell proliferation in CRC. AIM2‐deficient mice show increased gut microbial diversity, particularly the overgrowth of pathogenic bacteria, leading to enhanced susceptibility to inflammation and tumor formation. NLRC4 recognizes bacterial flagellin and endotoxins to activate pyroptosis, protecting IECs from pathogen invasion. In NLRC4‐deficient mice, colitis and tumor formation are significantly increased. Changes in the *Bacteroidetes*/*Firmicutes* ratio, particularly a reduction in *Firmicutes*, are commonly associated with increased inflammation and a higher risk of intestinal cancer. The overgrowth of pathogenic *Proteobacteria* such as *E. coli* and *Salmonella* is closely linked to excessive pyroptosis activation and the disruption of intestinal barrier function. Furthermore, the decrease in *A. muciniphila* and *Lactobacillus* is associated with weakened barrier function and increased inflammation, further promoting the overgrowth of pathogenic bacteria and the abnormal activation of pyroptosis.^67^, ^68^

Multiple studies have shown that various compounds and interventions regulate gut microbiota and pyroptosis‐related pathways in different gastrointestinal diseases. In DSS‐induced colitis, hydroxysafflor yellow A was found to increase *Proteobacteria* and *Escherichia–Shigella*, while reducing *Bacteroidetes* and *Enterococcaceae*, and inhibiting GSDMD‐mediated pyroptosis and NLRP3 inflammasome signaling pathways.^69^ In the same model, Kuijietong increased *Erysipelotrichia* and *Turicibacter* populations, decreased *Ruminococcaceae*, and inhibited NIMA‐related kinase 7 (NEK7)‐mediated pyroptosis and NLRP3 inflammasome signaling pathways.^70^ Salidroside regulated the *Firmicutes* and *Bacteroidetes* populations, inhibiting the NLRP3 inflammasome and TREM1 signaling pathways.^71^ Cyanidin‐3‐galactoside modulated *Proteobacteria* and *Verrucomicrobia* populations, suppressing NLRP3 inflammasome and associated inflammatory cytokine expression.^72^ MCC950, as an NLRP3 inhibitor, not only suppressed the NLRP3 inflammasome and related inflammatory factors but also altered the gut microbiota by increasing *Firmicutes* and reducing *Bacteroidota* and *Lachnospiraceae*.^73^

In MFC cell line‐induced gastric cancer, modified Gexia Zhuyu Decoction increased *Bacteroidaceae* and *Paraprevotellaceae* populations, and activated the NLRP3 inflammasome and related pyroptosis pathways.^74^ Early weaning‐induced intestinal inflammation and diarrhea were significantly alleviated by fecal microbiota transplantation, which reduced *Proteobacteria* and *Escherichia‐Shigella* levels, increased *Rikenellaceae_RC9_gut_group*, and inhibited NLRP3 inflammasome signaling and associated pyroptosis.^75^ In herbicide‐induced gut barrier damage, propisochlor led to an increase in *Bacteroidetes* and *Proteobacteria*, and activated the NLRP3 inflammasome and pyroptosis pathways.^76^ In chronic cold stress‐induced intestinal injury, a reduction in *Muribaculaceae* and *Lactobacillus* was observed, along with significant upregulation of NLRP3 inflammasome and mitogen‐activated protein kinase (MAPK) pathways.^77^ In aflatoxin B1 (AFB1)‐induced gut injury, curcumin modulated *Lactobacillus* and *Streptococcus* populations, promoting the activation of the NLRP3 inflammasome and TLR4/NF‐κB pathways.^78^ In 5‐fluorouracil‐induced intestinal mucositis, moxibustion increased *Lactobacillus* and *Roseburia* populations, activating the NLRP3 inflammasome and pyroptosis pathways.^79^ In azoxymethane and DSS‐induced CRC, an increase in *Lactobacillus* populations was observed, along with significant activation of the NLRP3 inflammasome pathway.^80^ Finally, in intestinal inflammation induced by long‐term ingestion of sunset yellow dye, increases in *Prevotella 2* and *Bilophila* populations were observed, with activation of NLRP3, TLR4/NLRP3/ASC/caspase‐1 pathways (Table 1).^81^

**TABLE 1:** Compilation of literature on the regulation of pyroptosis in diseases through the gut microbiota.

Disease	Disease model	Medium	Gut microbiota	Metabolite or molecular mechanism	References
Gastrointestinal disease	DSS‐induced colitis	Hydroxysafflor yellow A	*Proteobacteria* ↑ *Bacteroidetes* ↓ *Enterobacteriaceae* ↑ *Enterococcaceae* ↓ *Escherichia–Shigella* ↑ *Enterococcus* ↑	GSDMD‐mediated pyroptosis ↓ NLRP3 inflammasome signaling pathway ↓ HK1 expression ↓ IL‐1β ↓ IL‐6 ↓ TNF‐α ↓ IL‐18 ↓ Intestinal barrier function ↑	^69^
	DSS‐induced UC	Kui Jie Tong	*Erysipelotrichia* ↑ *Erysipelotrichales* ↑ *Erysipelotrichaceae* ↑ *Turicibacter* ↑ *uncultured_bacterium_g_Turicibacter* ↑ *Ruminococcaceae* ↓ *unclassified_f_Ruminococcaceae* ↓ *unclassified_g_Ruminococcus_1* ↓	NEK7‐mediated pyroptosis ↓ NLRP3 inflammasome signaling pathway ↓ Caspase‐1 ↓ GSDMD ↓ IL‐1β ↓ IL‐18 ↓ IL‐33	^70^
	Dextran sulphate sodium‐induced colitis	Salidroside	*Firmicutes* ↑ *Deinococcus‐Thermus* ↑ *Gemmatimonadetes* ↑ *Nitrospirae* ↑ *Bacteroidetes* ↓ *Akkermansia* ↓ *uncultured_bacterium_f_Muribaculaceae* ↓ *Lachnospiraceae* ↑ *Ruminococcaceae* ↑ *Escherichia‐Shigella* ↑ *Turicibacter* ↑	NLRP3 inflammasome signaling pathway ↓ GSDMD ↓ Caspase‐1 ↓ IL‐1β ↓ TREM1 signaling pathway ↓	^71^
	Dextran sulfate sodium‐induced colitis	Pelargonidin‐3‐galactoside	*Proteobacteria* ↓ *Deferribacteres* ↓ *Firmicutes* ↑ *Bacteroidetes* ↑ *Verrucomicrobia* ↑	NLRP3 inflammasome signaling pathway ↓ Cleaved‐Caspase‐1 ↓ IL‐6 ↓ IL‐1β ↓ TNF‐α ↓ N‐GSDMS	^72^
	DSS‐induced IBD	MCC950 (NLRP3 inhibitor)	*Firmicutes*↑ *Bacteroidota* ↓ *Lactobacillus*↑ *Lachnospiraceae* ↓	NLRP3 ↑ IL‐1β ↑ Caspase‐1 ↑ SOD ↑ Catalase ↑ MDA ↓	^73^
	MFC cell line‐induced gastric cancer	Modified Gexia‐Zhuyu Tang	*Bacteroidaceae* ↑ *Paraprevotellaceae* ↑ *Prevotella* ↑ *Psychrobacter* ↑ *Coprococcus* ↑ *Oscillospira* ↑ *Sutterella* ↑ *Alistipes* ↑	NLRP3 inflammasome ↑ Caspase‐1 ↑ TNF‐α ↑ IL‐1β ↑ IL‐18 ↑ LDH ↑	^74^
	Early weaning‐induced intestinal inflammation and diarrhea	Fecal microbiota transplantation	*Proteobacteria* ↓ *Escherichia‐Shigella* ↓ *Rikenellaceae_RC9_gut_group* ↑ *Ruminococcus* ↑	Pyroptosis suppression: NLRP3 ↓ Caspase‐1 ↓ GSDMD‐N ↓ Pro‐IL‐1β ↓ IL‐1β ↓ TLR4 ↓ ROS ↓ LPS ↓ Butyric acid ↑	^75^
	Herbicide‐induced intestinal barrier impairment	Propisochlor	*Bacteroidetes* ↑ *Firmicutes* ↓ *Proteobacteria* ↑ *Parabacteroides* ↑ *Parasutterella* ↑ *Bacteroides* ↑ *Alistipes* ↑ *Desulfovibrionaceae* ↑ *Staphylococcus* ↓ *Jeotgalicoccus* ↓	NLRP3 inflammasome signaling pathway ↑ TLR4 ↑ Caspase‐1 ↑ GSDMD ↑ IL‐1β ↑ IL‐18 ↑ LPS ↑ ZO‐1 ↓ Occludin ↓ Claudin‐1 ↓ MUC2 ↓	^76^
	Chronic cold stress‐induced intestinal injury		*norank_f_Muribaculaceae* ↓ *Lactobacillus* ↓ *Carnobacterium* ↓ *Romboutsia* ↑ *Corynebacterium* ↑ *Turicibacter* ↑ *Muribaculum* ↑	NLRP3 inflammasome signaling pathway ↑ GSDMD ↑ Caspase‐1 ↑ Caspase‐11 ↑ IL‐1β ↑ IL‐18 ↑ TLR4/MyD88–NF‐κB signaling pathway ↑ MAPK signaling pathway ↑	^77^
	AFB1‐induced intestinal injury	Curcumin	*Lactobacillus* ↓ *Streptococcus* ↓ *Enterococcus* ↓ *Actinobacteria* ↑ *Bacteroidetes* ↑ *Bacteroidales* ↑ *Clostridiales* ↑ *Corynebacteriales* ↓	NLRP3 inflammasome signaling pathway ↑ GSDMD ↑ Caspase‐1 ↑ IL‐1β ↑ IL‐18 ↑ TLR4/NF‐κB signaling pathway ↑Pyroptosis ↑	^78^
	5‐Fluorouracil‐induced intestinal mucositis	Moxibustion	*Lactobacillus* ↑ *Roseburia* ↑ *Escherichia* ↑ *Akkermansia* ↑	NLRP3 ↑ Caspase‐1 ↑ GSDMD↑ Pyroptosis ↑ IL‐18 ↑ IL‐10 ↓	^79^
	Azoxymethane and DSS‐induced colon cancer		*Lactobacillus* ↑ *Alloprevotella* ↓ *Desulfovibrionaceae* ↓	NLRP3 ↑ Caspase‐1↑ GSDMD ↑	^80^
	Chronic consumption of Sunset Yellow dye‐induced intestinal inflammation	Sunset Yellow dye	*Treponema 2* ↓ *Anaerobiospirillum* ↓ *Helicobacter* ↓ *Rikenellaceae RC9 gut group* ↓ *Prevotellaceae UCG‐003* ↓ *Prevotella 2* ↑ *Oribacterium* ↑ *Bilophila* ↑ *Mailhella* ↑ *Faecalibacterium* ↑ *GCA‐900066225* ↑	NLRP3 inflammasome signaling pathway ↑ TLR4/NLRP3/ASC/caspase‐1 pathway ↑ Caspase‐11 ↑ GSDM‐N ↑ IL‐1β ↑ IL‐18 ↑	^81^
Liver disease	S100 antigen emulsified with Freund's adjuvant to induce autoimmune hepatitis	Synbiotic	*Rikenella* ↑ *Alistipes* ↑ *Dubosiella* ↑ *unclassified_g_Lachnospiraceae_NK4A136_group* ↑ *unclassified_f_Lachnospiraceae* ↑ *Escherichia‐Shigella* ↓ *Lactobacillus* ↓ *Proteobacteria* ↓ *Patescibacteria* ↓ *Deferribacterota* ↓ *Enterobacteriaceae* ↓ *norank_o_Clostridia_UCG‐014* ↓	NLRP3 inflammasome signaling pathway ↓ TLR4/NF‐κB signaling pathway ↓ LPS ↓ Caspase‐1 ↓ IL‐1β ↓ ZO‐1 ↑ Occludin ↑	^114^
	Polyethylene microplastics‐induced liver injury	Polyethylene microplastics	*Desulfovibrio* ↑ *Clostridia* ↑ *Enterorhabdus* ↑ *Bacteroides* ↑ *Gemella* ↑ *Dubosoella* ↓	TLR2/NF‐κB/NLRP3 signaling pathway ↑	^115^
	High‐fat diet‐induced NAFLD	Xie Zhuo Tiao Zhi decoction	*Ileibacterium valens* ↑ *Bifidobacterium pseudolongum* ↑ *Akkermansia muciniphila* ↑ *Faecalibacterium rodentium* ↓ *Lactobacillus reuteri* ↓	Purine metabolism ↑ Fatty acid β‐oxidation ↑ Stearic acid biosynthesis ↓ Arachidonic acid metabolism ↓ Linoleic acid metabolism ↓ Inflammatory cytokines (IL‐6, TNF‐α, IL‐1β) ↓ Pyroptosis‐related proteins (NLRP3, GSDMD‐N, Nek7, ASC, Caspase‐1 p20) ↓	^116^
	Huangjiu‐induced liver injury	Histamine	*norank_f__Muribaculaceae* ↓ *Prevotellaceae_UCG‐001* ↑ *Lachnospiraceae_NK4A136_group* ↓ *Pseudogracbacilus* ↑ *Sporosarcina* ↓ *Firmicutes* ↑ *Bacteroides* ↑ *Proteobacteria* ↓	LPS ↑ Caspase‐1 ↑ Inflammatory cytokines (IL‐1β, Cas‐1, GSDMD) Pyroptosis pathway activation ↑	^117^
	Alcohol‐induced liver injury	Compound probiotic	*Firmicutes* ↑ *Bacteroidota* ↓ *Proteobacteria* ↓ *Actinobacterota* ↑ *Verrucomicrobiota* ↑ *Desulfobacterota* ↓ *Lachnospiraceae* ↑ *Lactobacillaceae* ↑ *Enterobacteriaceae* ↓ *Prevotellaceae* ↓ *Akkermansiaceae* ↑ *Bifidobacteriaceae* ↑	TLR4/NF‐κB signaling pathway ↓ LPS ↓	^118^
	Alcoholic fatty liver disease	Butyrate	*Parabacteroides* ↓ *Murbaculum* ↓ *Cyanobacteria* ↓ *Klebsiella* ↓ *Bacteroidetes* ↓ *Firmicutes* ↓ *Proteobacteria* ↓ *Verrucomicrobia* ↑ *Actinobacteria* ↑	GSDMD‐mediated pyroptosis ↓ NLRP3 inflammasome signaling pathway ↓ IL‐1β ↓ IL‐18 ↓ ZO‐1 ↑ LPS ↓	^119^
	High‐fat and high‐fructose diet‐induced nonalcoholic fatty	Lycopene	*Firmicutes* ↓ *Lachnospiraceae_NK4A136_group* ↓ *Desulfovibrio* ↓ *Alistipes* ↓ *Alloprevotella* ↑ *Blautia* ↑ *Verrucomicrobia* ↑ *Firmicutes* ↓ *Proteobacteria* ↓	NLRP3 inflammasome signaling pathway ↓ TLR4/NF‐κB signaling pathway ↓ TNF‐α ↓, IL‐6 ↓ Caspase‐1 ↓ IL‐1β ↓	^120^
	AFB1 exposure‐induced liver injury		*Bacteroides* ↑ *Parabacteroides* ↑ *Lactobacillus* ↑ *Escherichia‐Shigella* ↑ *Alistipes* ↓	TLR4/NLRP3/caspase‐1 signaling pathway ↑ Liver pyroptosis ↑ Colonic barrier dysfunction ↑ LPS ↑	^121^
	AFB1‐induced liver pyroptosis		*Helicobacter* ↑ *Bacteroides* ↑ *Lactobacillus* ↑ *Alistipes* ↑ *Parabacteroides* ↑ *Escherichia‐Shigella* ↑ *Ileibacterium* ↑ *Akkermansia* ↑	TLR4/NLRP3 signaling pathway ↑ Caspase‐1 ↑ IL‐1β ↑ IL‐18 ↑ Pipecolic acid ↑ Norepinephrine ↑	^122^
	Aging‐induced liver injury	Ginsenoside Rg2	*Firmicutes* ↑ *Bacteroidota* ↓ *Proteobacteria* ↓ *Actinomycetes* ↓ *Lactobacillus* ↑ *norank_f_Muribaculaceae* ↑ *Staphylococcus* ↓ *Lachnospiraceae_NK4A136_group* ↓	Caspase‐8‐mediated pyroptosis pathway ↓ NLRP3 inflammasome signaling pathway ↓ GSDMD‐N ↓ IL‐1β ↓ IL‐18 ↓ TNF‐α ↓	^123^
	Zinc oxide nanoparticles‐induced hepatotoxicity	Paeoniflorin	*Proteobacteria* ↑ *Firmicutes* ↑ *Verrucomicrobiota* ↑ *Lactobacillus* ↑ *Akkermansia* ↑ *Desulfovibrio* ↑ *Prevotellaceae* ↑ *Rikenellaceae* ↑ *Escherichia‐Shigella* ↓ *Enterococcus* ↓ *Bacteroides* ↓	SIRT1–mTOR–TFEB axis ↓ NLRP3 inflammasome signaling pathway ↓ Caspase‐1 ↓ IL‐18 ↓ GSDMD‐N ↓	^124^
	Terbuthylazine‐induced hepatotoxicity		*Firmicutes* ↓ *Nitrospirota* ↑ *Chloroflexi* ↑ *Desulfobacterota* ↑ *Crenarchaeota* ↑ *Myxococcota* ↑ *Planctomycetota* ↑	Mitochondrial quality control imbalance ↑ Necroptosis ↑ Occludin ↓ ZO‐1 ↓	^125^
	High‐fat diet and CCl₄‐induced liver fibrosis	Corn gluten‐derived sEH inhibitory peptides	*Firmicutes/Bacteroidetes* ↓ *Monoglobust* ↑ *Olsenellat* ↑	NLRP3 inflammasome suppression: NLRP3 ↓ ASC ↓ SCFAs ↑ Pro‐caspase‐1 ↓ Caspase‐1 ↓ GSDMD ↓ Pro‐IL‐1β ↓ Pro‐IL‐18 ↓	^126^
	Methionine choline deficiency high‐fat diet‐induced nonalcoholic steatohepatitis	Parabacteroides distasonis	*Escherichia‐Shigella* ↓ *Enterobacter* ↓ *Lactobacillus* ↑	NLRP3 inflammasome suppression: NLRP3 ↓ ASC ↓ Caspase‐1 ↓ AhR pathway activation: AhR ↑ Indoleacetic acid ↑ IL‐1β ↓ IL‐18 ↓	^127^
	Bisphenol A‐induced liver injury	Geraniol	*Bacteroidetes* ↑ *Actinobacteria* ↑ *Verrucomicrobia* ↓ *Clostridium XlVa* ↑ *Ruminococcus* ↑ *Alistipes* ↓ *Clostridium IV* ↑ *unclassified_Firmicutes* ↑ *unclassified_Ruminococcaceae* ↑ *Prevotella* ↓	NLRP3/Caspase‐1 signaling pathway ↓ ASC ↓ ZO‐1 ↑ Claudin‐1 ↑ Occludin ↑	^128^
	High‐fat and high‐cholesterol diet‐induced NAFLD exacerbated by nicotine	Bacteroides xylanisolvens	*Bacteroides xylanisolvens* ↑ *Bacteroidota (including Bacteroides spp.)* ↓ *Firmicutes* ↓ *Actinomycetes* ↓	4‐Hydroxy‐1‐(3‐pyridyl)‐1‐butanone (HPB) ↑ Nicotine accumulation in the intestine activates AMPKα → increased phosphorylation of SMPD3 → elevated ceramide production → enhanced NASH progression.	^129^
	Ethanol‐induced liver steatosis and inflammation	Diallyl disulfide	*Rikenella* ↑ *Alistipes* ↑ *Dubosiella* ↑ *unclassified_g_Lachnospiraceae_NK4A136_group* ↑ *unclassified_f_Lachnospiraceae* ↑ *Escherichia‐Shigella* ↓ *Lactobacillus* ↓ *Proteobacteria* ↓ *Patescibacteria* ↓ *Deferribacterota* ↓ *Enterobacteriaceae* ↓ *norank_o_Clostridia_UCG‐014* ↓	NLRP3 inflammasome signaling pathway ↓ NF‐κB signaling pathway ↓ LPS ↓ Caspase‐1 ↓ IL‐1β ↓ ZO‐1 ↑ Occludin ↑	^130^
	Concanavalin A‐induced liver cirrhosis	Splenectomy	*Veillonella* ↑ *Bacteroides* ↓ *Parabacteroides* ↓ *Paraprevotella* ↓ *Odoribacter* ↓	TLR4/NLRP3 signaling pathway ↓ IL‐1β ↓ IL‐6 ↓ TNF‐α ↓	^131^
Cerebral disease	Aging‐induced intestinal barrier dysfunction	Bazi Bushen capsule	*Firmicutes* ↑ *Ruminococcaceae* ↑ *Lachnospiraceae* ↑ *Ruminnococcaceae_UCG_014* ↑ *Ruminnococcaceae_UCG_004* ↑ *Ruminiclostridium (various strains)* ↑ *Oscillibacter* ↑ *Neglecta* ↑ *Intestinimonas* ↑ *Peptococcus* ↑ *Roseburia* ↑ *Eubacterium_xylanophilum_group* ↑ *Anaerotignum* ↑ *Acetatifactor* ↑ *Eubacterium_brachy_group* ↑	NLRP3 inflammasome signaling pathway ↓ Caspase‐1 ↓ IL‐1β ↓ IL‐18 ↓ GSDMD ↓ LPS ↓	^136^
	Low, high, and variable temperature‐induced GBA dysfunction	Temperature stress (low, high, variable)	*Low temperature (LT)*: *Bacteroidetes* ↑ *Verrucomicrobia* ↑ *Muribaculaceae* ↑ *Rikenellaceae* ↑ *Prevotellaceae* ↑ *Ruminococcaceae* ↑ *Lactobacillaceae* ↓ *High temperature (HT)*: *Actinobacteria* ↑ *Prevotellaceae* ↑ *Peptostreptococcaceae* ↑ *Ruminococcaceae* ↑ *Temperature variation (TV)*: *Actinobacteria* ↓ *Verrucomicrobia* ↓ *Rikenellaceae* ↑ *Bacteroidaceae* ↑ *Bifidobacteriaceae* ↑ *Akkermansiaceae* ↑	Low temperature (LT): NLRP3 inflammasome ↓ IL‐1β ↓ ASC ↓ Caspase‐1 ↓ High temperature (HT): NLRP3 inflammasome ↑ IL‐1β ↑ ASC ↑ Caspase‐1 ↑ IL‐6 ↑ TNF‐α ↑ Temperature variation (TV): NLRP3 inflammasome ↑ TNF‐α ↑	^137^
	LPS‐induced sepsis‐associated encephalopathy	NU9056(a KAT 5 Inhibitor)	*Verrucomicrobia* ↑ *Akkermansia* ↑ *Lachnoclostridium* ↑ *Alloprevotella* ↓ *Parabacteroides* ↓ *Bacteroides* ↓ *Escherichia‐Shigella* ↓ *Roseburia* ↓ *Lachnospiraceae_NK4A136_group* ↓	NLRP3 inflammasome ↓ ASC ↓ Caspase‐1 ↓ P10 ↓ IL‐1β ↓ IL‐6 ↓ SCFAs ↑ (Acetate, propionate, butyrate)	^138^
	5xFAD‐induced AD		*Firmicutes* ↓ *Bacteroidetes* ↑ *Bifidobacteria* ↓ *Lactobacillus* ↓	NLRP3 inflammasome ↑ ASC ↑ Caspase‐1 ↑ IL‐1β ↑ GSDMD ↑	^139^
	3.5 GHz RFR‐induced neuronal pyroptosis and anxiety‐like behavior		*Lactobacillaceae* ↑ *norank_o__Clostridia_UCG‐014* ↑ *Saccharimonadaceae* ↑ *Muribaculaceae* ↓ *Rikenellaceae* ↓ *Lachnospiraceae* ↓ *Eggerthellaceae* ↓ *Bacteroidaceae* ↓ *Marinifilaceae* ↑ *Ruminococcaceae* ↑ *Monoglobaceae* ↑	NLRP3 inflammasome ↑ GSDMD ↑ Cleaved caspase‐1 ↑	^140^
	Chronic unpredictable mild stress	Xiaoyaosan	*Bacteroidetes/Firmicutes ratio* ↓ *Bacteroides* ↓ *Corynebacterium* ↓ *Lactobacillus* ↓ *Adlercreutzia* ↓	LPS ↓ TLR4 ↓ NLRP3 ↓ NF‐κB ↓ IL‐1β↓ Claudin 1 ↓ ZO‐1 ↓	^141^
	Manganese chloride‐induced neurotoxicity	Fecal microbiota transplantation	*Clostridiales* ↑ *Pseudoflavonifractor* ↑ *Ligilactobacillus* ↑ *Desulfovibrio* ↑ *Anaerotruncus* ↓ *Eubacterium_ruminantium_group* ↓ *Fusimonas* ↓ *Firmicutes* ↓	cGAS–STING pathway ↓ NLRP3 inflammasome signaling pathway ↓	^142^
	Chronic unpredictable mild stress‐induced neurotoxicity	Probiotic administration (Lactobacillus rhamnosus HN001, Bifidobacterium animalis subsp. lactis HN019)	*Actinobacteria* ↓ *Proteobacteria* ↓ *Patescibacteria* ↓ *Lactobacillaceae* ↑ *Erysipelotrichaceae* ↑ *Lachnospiraceae* ↑ *Lactobacillus* ↓ *Lachnospiraceae NK4A136 group* ↑	NLRP3 inflammasome signaling pathway ↓ IL‐1β ↓ IL‐18 ↓	^143^
	Chronic sleep deprivation‐induced neurotoxicity	Sleep deprivation microbiota transplantation	*Bacteroidetes* ↑ *Proteobacteria* ↓ *Firmicutes* ↓ *Odoribacter* ↑ *Lactobacillus* ↓	NLRP3 inflammasome signaling pathway ↑ Autophagy dysfunction ↑ Tau hyperphosphorylation ↑ GSK‐3β activation ↑	^144^
Cardiovascular disease	Age‐related AF in aged rats and humans	Fecal microbiota transplantation	*Firmicutes* ↓ *Bacteroidetes* ↑ *Spirochaetae* ↑ *Verrucomicrobia* ↓ *Lactobacillus spp*. ↓ *Bacteroidaceae* ↑	NLRP3 inflammasome activation ↑ TLR4/NFκB pathway activation ↑ LPS ↑ Glucose ↑ Atrial fibrosis ↑ IL‐1β ↑ TGF‐β1 ↑ α‐SMA ↑	^147^
	Cold exposure‐induced AF	Akkermansia muciniphila		TMAO↑ Caspase‐1 ↑ Cleaved‐GSDMD ↑ TGF‐β1 ↑ α‐SMA ↑	^148^
	Angiotensin II and β‐aminopropionitrile fumarate‐induced aortic dissection		*Escherichia‐Shigella* ↑ *Alistipes* ↑ *Parabacteroides* ↑ *Klebsiella* ↑ *Akkermansia* ↑	NLRP3 ↑ ASC ↑ Caspase‐1 ↑ TLR4	^149^
Other diseases	CLP‐induced sepsis model	Qi Huang Fang	*Firmicutes* ↑ *Muribaculaceae* ↑ *Campilobacterota* ↑ *Helicobacter* ↑ *Alistipes* ↑ *Bacteroidetes* ↑ *Actinobacteria* ↑	GSDMD‐mediated pyroptosis ↓ NLRP3 inflammasome signaling pathway ↓ Caspase‐1 ↓ Caspase‐11 ↓ IL‐6 ↓ IL‐1β ↓ TNF‐α ↓ ZO‐1 expression ↑ Occludin expression ↑	^150^
	CLP‐induced sepsis	Fecal microbiota transplantation and SCFAs	*Firmicutes* ↓ *Proteobacteria* ↑ *Escherichia‐Shigella* ↑ *Lactobacillus* ↑	NLRP3 ↓ GSDMD‐N ↓ IL‐1β ↓ IL‐18 ↓	^151^
	Pregnancy‐induced sepsis	Parabacteroides merdae		FMN–hnRNPUL2–NLRP3 signaling axis ↑ Caspase‐1 ↑ IL‐1β ↑ IL‐18 ↑ Macrophage pyroptosis ↑	^152^
	AFB1‐induced immunotoxicity	AFB1	*Bacteroidota/Firmicutes ratio* ↓ *Firmicutes* ↑ *Lactobacillus* ↑ *Dubosiella* ↑ *Parabacteroides* ↑ *Alistipes* ↓ *Helicobacter* ↓ *Eubacterium* ↓	LPS ↑ TLR4/NF‐κB/NLRP3 signaling pathway ↑ Pyroptosis (IL‐1β, IL‐18, Caspase‐1, GSDMD)↑	^153^
	DHEA‐induced PCOS	Disulfiram and Metformin	*Akkermansia* ↓ *Desulfovibrio* ↑ *Burkholderia* ↑	GSDMD ↓ Caspase‐1 ↓ LPS ↓ IFN‐γ ↓ Estrogen synthesis disruption ↓ Granulosa cells apoptosis ↑	^154^
	High‐fat diet‐induced metabolic endotoxemia		*Proteobacteria* ↑ *Deferribacterota* ↓ *Desulfobacterota* ↓ *Campilobacterota* ↓ *Firmicutes/Bacteroidota ratio* ↓ *Actinobacteriota* ↑ *Patescibacteria* ↑	GSDMD‐N/CL binding ↑ Caspase‐1 ↑ IL‐1β ↑ LPS ↑ GSDMD‐N killing proteobacteria ↑ Gut permeability ↓ (ZO‐1, Occludin, Claudin)	^155^

### 5.2 Mechanisms of apoptosis in intestinal flora and gastrointestinal diseases

Increased apoptosis leading to epithelial barrier disruption is considered a key factor in the pathogenesis of IBD, including CD and UC. In UC, enhanced apoptosis of epithelial cells weakens the intestinal barrier, allowing bacteria and immune cells to penetrate, thereby exacerbating inflammation. In contrast, in CD, apoptosis of immune cells such as T cells is often suppressed, prolonging their survival and contributing to chronic inflammation.^82^, ^83^ Certain bacteria, such as *Salmonella dublin*, *Yersinia enterocolitica*, *Shigella dysenteriae*, and *Listeria monocytogenes*, induce epithelial cell apoptosis by stimulating the secretion of proinflammatory cytokines such as TNF, leading to epithelial damage and worsening mucosal inflammation.^84^ Sulfate‐reducing bacteria, particularly *Desulfovibrio indonesiensis*, negatively impact UC by inducing apoptosis in IECs. In experiments, *D. indonesiensis* was internalized by epithelial cells, both in mono‐culture and coculture with *E. coli*, and induced apoptosis. In UC patients, sulfate‐reducing bacteri (SRB) populations significantly enhanced this apoptotic effect, while this was not observed in healthy controls. Additionally, antibodies against the polysaccharides of *D. indonesiensis* cross‐reacted with the SRB population in UC patients, but not in healthy individuals.^85^ In inflamed intestinal tissues of UC patients, researchers identified *Fusobacterium varium*, whose culture supernatant contained high levels of butyrate, which was cytotoxic to Vero cells. Further animal experiments showed that mice receiving enemas containing butyrate or *F. varium* supernatant developed UC‐like lesions, including crypt abscesses, inflammatory cell infiltration, and apoptotic changes.^86^ Conversely, studies have shown that coculturing *Lactobacillus casei* with intestinal mucosa significantly reduced the release of proinflammatory cytokines, decreased T lymphocyte activation in the lamina propria, and reduced the expression of the antiapoptotic protein Bcl‐2, increasing T cell apoptosis, potentially aiding in the restoration of immune homeostasis.^87^ Specific probiotics, such as *Lactobacillus rhamnosus GG*, protected the epithelial barrier by activating antiapoptotic signaling pathways, such as the Akt/protein kinase B (PKB) pathway, and inhibiting proapoptotic pathways like p38 kinase (p38)/MAPK, thereby reducing TNF‐induced epithelial cell apoptosis.

In studies on the effects of gut microbiota on distal organs, the relationship between gut microbiota and ferroptosis or pyroptosis is stronger than its connection to apoptosis. Ferroptosis is an iron‐dependent form of cell death triggered by lipid peroxidation, and the gut microbiota plays a critical role by regulating host iron and lipid metabolism. Certain gut bacteria can influence iron levels and the generation of lipid peroxidation products, thereby inducing ferroptosis. Additionally, pathogens such as *Pseudomonas aeruginosa* can directly induce ferroptosis in host cells by producing enzymes like lipoxygenase.^88^, ^89^

Pyroptosis, a form of cell death dependent on inflammasomes, also shows a strong connection with gut microbiota. LPS is widely recognized as a potent trigger for the activation of the NLRP3 inflammasome.^90^ This process plays a significant role in gut inflammatory diseases.

In contrast, apoptosis is a noninflammatory form of cell death. Compared with pyroptosis, gut microbiota often induces apoptosis indirectly through the TNF‐α pathway, or by causing mitochondrial dysfunction or disruptions due to environmental factors. These processes typically trigger apoptosis in a more secondary manner, relying on the interplay of inflammatory signals or stress conditions within the cellular environment.^91^ Therefore, this paper will not delve into the effect of intestinal flora on distal organ apoptosis.

### 5.3 Mechanisms of ferroptosis in intestinal flora and gastrointestinal diseases

The gut microbiota significantly impacts ferroptosis through various mechanisms, playing a crucial role in gut diseases. For example, *P. aeruginosa* can induce ferroptosis by producing a bacterial 15‐lipoxygenase that specifically oxidizes arachidonic acid phosphatidylethanolamin in host cells. This oxidation leads to the formation of 15‐hydroperoxy‐arachidonoyl‐P, a key lipid peroxidation product that triggers ferroptotic cell death, particularly in epithelial cells. This mechanism has been documented in both bronchial epithelial cells and gut epithelial cells under conditions of infection by *P. aeruginosa*, contributing to the pathogenesis observed during infections.^92^ This process, termed “theft‐ferroptosis,” highlights the bacteria's ability to hijack host lipid metabolism, facilitating oxidative damage and ferroptotic signaling, which may be exacerbated in immunocompromised conditions or during irradiation.^88^ In contrast, *E. coli* mitigates oxidative stress and inhibits ferroptosis by breaking down H_2_O_2_ and assimilating Fe^2+^.^93^ Probiotics such as *L. rhamnosus* GG help restore gut microbiota balance and reduce ferroptosis by upregulating NADPH oxidase 1 (NOX1) expression and activating the nuclear factor erythroid 2‐related factor 2 (NRF2) pathway to suppress oxidative stress.^94^ Additionally, microbial metabolites like glycochenodeoxycholic acid promote lipid peroxidation and ferroptosis by activating the Transferrin receptor and long‐chain acyl‐CoA synthetase 4 (TfR–ACSL4) pathway, while lactic acid‐producing bacteria inhibit ferroptosis by reducing the toxicity of PUFAs through saturation regulation. Dysbiosis of the gut microbiota contributes to CRC by increasing ROS/reactive nitrogen species (RNS) production, causing DNA damage, oxidative stress, and chromosomal instability, and promoting tumorigenesis via macrophage‐released TNF‐α. In IBD, ferroptosis exacerbates inflammation due to oxidative stress, and inhibiting ferroptosis or lipoxygenase enzymes can alleviate DSS‐induced colitis symptoms. Last, in intestinal ischemia–reperfusion injury (IRI), gut microbiota metabolites like capsiate activate the transient receptor potential vanilloid 1 (TRPV1) pathway, enhance GPX4 expression, reduce ferroptosis, and mitigate IRI symptoms.^95^

In gastrointestinal disease research, the effects of different mediums on gut microbiota and ferroptosis‐related molecular mechanisms have been extensively studied. For instance, in ischemia/reperfusion (I/R)‐induced intestinal injury, capsiate medium reduces MDA and Fe^2^⁺ levels while increasing GSH levels and the GSH/glutathione disulfide (GSSG) ratio by upregulating TRPV1 and GPX4 expression. This modulates the gut microbiota, leading to an increase in *Firmicutes* and *Bacteroidetes* and a decrease in *Verrucomicrobia*.^96^ Fluoride exposure, however, induces colonic ferroptosis by mediating N6‐methyladenosine (m6A) silencing of SLC7A11 and accelerating its degradation through YTH N6‐methyladenosine RNA binding protein 2 (YTHDF2) binding. This leads to iron overload, reduced GSH, and increased ROS, along with an increase in *Lactobacillus murinus*, a reduction in *Firmicutes*, and an increase in *Bacteroidetes*.^97^

An iron‐overloaded diet can result in iron‐overload‐induced colitis, with mechanisms involving the downregulation of the NRF2/GPX4 signaling pathway, leading to elevated MDA, ROS, and Fe^2^⁺ levels. A reduction in *Bifidobacterium* and *Lactobacillus*, along with an increase in *Akkermansia*, has also been observed.^98^ Early gut microbiota depletion exacerbates DSS‐induced colitis, marked by a reduction in anaerobes like *Rikenella* and *Alistipes*, along with decreased GPX4 and increased cyclooxygenase‐2 (COX‐2) and MDA levels.^99^ Treatment with deferasirox for DSS‐induced colitis reduces oxidative stress, upregulates ferroptosis markers GPX4 and ferritin heavy chain (FTH), and promotes the production of SCFAs such as butyrate, valerate, isobutyrate, and isovalerate.^100^

Other studies have shown that phlorizin and hesperetin alleviate DSS‐induced colitis by reducing iron overload and lipid peroxidation through the regulation of GPX4 and COX‐2. This effect is accompanied by an increase in beneficial bacteria, such as *Lactobacillaceae* and *Prevotellaceae*.^101^, ^102^ Amygdalin and anthocyanin‐rich cranberry extract reduce iron content and lipid peroxidation by upregulating antioxidant molecules like GPX4, FSP1, and NRF2, while increasing beneficial bacteria such as *S24‐7*, *Akkermansia*, and *Lactobacillus*.^103^, ^104^ Sodium butyrate has also been shown to reduce ferroptosis by activating the NRF2 pathway and downregulating ACSL4 and COX‐2, while increasing the proportion of *Proteobacteria* and inhibiting bacteria such as *Ruminococcaceae* (Table 2).^105^

**TABLE 2:** Compilation of literature on the regulation of ferroptosis in diseases through the gut microbiota.

Disease	Disease model	Medium	Gut microbiota	Metabolite or molecular mechanism	References
Gastrointestinal disease	I/R‐induced intestinal injury	Capsiate	*Firmicutes* ↑ *Bacteroidetes* ↑ *Verrucomicrobia* ↓ *Bacteroides vulgatus* ↑ *Parabacteroides distasonis* ↑	TRPV1 ↑ Gpx4 ↑ MDA ↓ Fe^2+^ ↓ Fth1 ↑ Cox‐2 ↓ GSH ↑ GSH/GSSG ↑	^96^
	Sodium fluoride‐induced colonic ferroptosis	Fluoride exposure	*Lactobacillus murinus (ASV54, ASV58, ASV82)* ↑ *Ileibacterium* ↓ *Firmicutes* ↓ *Bacteroidetes* ↑ *Proteobacteria* ↓ *Actinobacteria* ↑	Ferroptosis mediated by m6A silencing of SLC7A11: m6A modifications YTHDF2 binding ↑ SLC7A11 degradation ↑ GPX4 ↓ ROS ↑ Iron overload ↑ GSH ↓	^97^
	Iron overload‐induced colitis	High iron diet	*Dubosiella* ↓ *Lactobacillus* ↓ *Bifidobacterium* ↓ *Alloprevotella* ↑ *Romboutsia* ↑ *Akkermansia* ↑	NRF2/GPX4 signaling pathway ↓ MDA ↑ ROS ↑ C11‐BODIPY ↑ Fe^2+^ ↑	^98^
	DSS‐induced colitis	Early‐life microbiota depletion	*Rikenella* ↓ *Alistipes* ↓ *Lachnospiraceae_NK4A136_group* ↓ *Anaerobic bacteria* ↓ *Plasmalogen‐positive species* ↓ *Clostridia_UCG‐014* ↓	GPX4 ↓ COX‐2 ↑ MDA ↑ 4‐HNE ↑	^99^
	DSS‐induced colitis	Deferasirox	*Escherichia‐Shigella* ↓ *Streptococcus* ↓ *Dubosiella* ↑ *Lachnospiraceae_NK4A136_group* ↑ *Prevotellaceae_UCG‐001* ↑ *Odoribacter* ↑ *Blautia* ↑	GPX‐4 ↑ FTH ↑ ROS ↓ Fe^2+^ ↓ TF ↓ Butyric acid ↑ Valeric acid ↑ Isobutyric acid ↑ Isovaleric acid ↑	^100^
	DSS‐induced colitis	Phlorizin	*Lactobacillaceae* ↑ *Muribaculaceae* ↑ *Lachnospiraceae* ↓ *Firmicutes* ↑ *Bacteroidetes* ↓	Gpx4 ↑ MDA ↓ Iron load ↓ GSH ↑ FTH ↓ FTL ↓	^101^
	DSS‐induced colitis	Hesperetin	*Lachnospiraceae_NK4A136_group* ↑ *Prevotellaceae_UCG‐001* ↑ *Lachnospirales* ↑ *Oscillospirales* ↑ *Proteobacteria* ↓ *Gammaproteobacteria* ↓	PTGS2 ↓ ACSL4 ↓ Gpx4 ↑ MDA ↓ SOD ↑ Lipid peroxidation ↓	^102^
	DSS‐induced colitis	Amygdalin	*S24‐7* ↑ *Akkermansia* ↑ *Prevotella* ↑ *Ruminococcus* ↑ *Firmicutes* ↑ *Bacteroides* ↓ *Allobaculum* ↓ *Oscillospira* ↓ *Sutterella* ↓ *Erysipelotrichaceae* ↓	MDA ↓ GPX4 ↑ FSP1 ↑ xCT ↑ Nrf2 ↑ Iron content ↓ COX‐2 ↓	^103^
	DSS‐induced colitis	Anthocyanins‐rich cranberry extract	*Proteobacteria* ↓ *Escherichia‐Shigella* ↓ *Lactobacillus* ↑ *Bacteroidota* ↑ *Firmicutes* ↑	MDA ↓ GSH ↑ GPX4 ↑ SLC7A11 ↑ HO‐1 ↑ IL‐1β, IL‐6, TNF‐α ↓	^104^
	DSS‐induced colitis	Sodium butyrate	*Proteobacteria* ↑ *Deltaproteobacteria* ↑ *Desulfovibrionaceae* ↑ *Ruminococcaceae* ↓ *Bacteroidaceae* ↓ *Bacteroides* ↓	Nrf2 ↑ GPX4 ↑ ACSL4 ↓ COX2 ↓ FTH1 ↑ MDA ↓ Iron ↓ ROS ↓	^105^
Liver disease	Western diet‐induced NASH	Obeticholic acid	*Bacteroides* ↑ *Rikenellaceae* ↑ *Helicobacteraceae* ↑ *Lachnospiraceae* ↓ *Desulfobacterota* ↓ *Firmicutes* ↓	ARA‐derived 12‐HHTrE ↑ ROS ↑ MDA ↑ SLC7A11 ↓ GPX4 ↓ Lipid peroxidation ↑ TGF‐β1 ↑ COL1A1 ↑	^161^
	Bromoacetic acid‐induced MAFLD	Glycochenodeoxycholate		TFR–ACSL4 signaling ↑ GSH ↓ ROS ↑ iron ↑	^162^
	Porphyromonas gingivalis‐induced nonalcoholic fatty liver disease	Porphyromonas gingivalis	*Bacteroidetes* ↑ *Helicobacter* ↑ *Prevotella* ↑ *Coprococcus* ↓ *Desulfovibrio* ↓ *Firmicutes* ↓	GPX4 ↓ SLC7A11 ↓ ACSL4 ↑ Th17/Treg imbalance ↑	^163^
	CLP‐induced septic liver injury	Nobiletin	*Ligilactobacillus* ↑ *Akkermansia* ↑ *Lactobacillus* ↑ *Dubosiella* ↓ *Bacteroides* ↓	Nrf2–Gpx4 signaling ↑ GSH ↑ ROS ↓ iron metabolism ↓	^164^
	High‐fat diet‐induced steatotic liver transplantation	Fer‐1	*Peptostreptococcaceae* ↑ *Muribaculaceae* ↑ *Prevotellaceae* ↑ *Enterobacteriaceae* ↓ *Fusobacteriaceae* ↓ *Bacteroidaceae* ↓ *Lachnospiraceae* ↑ *Lactobacillaceae* ↑ *Oscillospiraceae* ↑	GPX4 ↑ Lipid ROS ↓ Iron metabolism regulation ↑ Butyrate ↑	^165^
	CDAHFD‐induced NAFLD	Urolithin C	*Firmicutes/Bacteroidota ratio* ↓ *Helicobacter ganmani* ↓ *Bacteroides caecimuris* ↓ *Lachnospiraceae bacterium DW59* ↓ *Enterorhabdus* ↓ *Lachnospiraceae bacterium DW46* ↓ *Alistipes sp. cv1* ↓ *Clostridiales bacterium CIEAF 021* ↓ *Clostridium sp. Culture‐41* ↓ *Parabacteroides goldsteinii* ↑ *Lactobacillus vaginalis* ↑	AMPK signaling pathway ↑ MUFAs ↑ Lipid peroxidation (4‐HNE) ↓, GPx2 ↑ ZO‐1 ↑ Occludin ↑	^166^
	Methionine–choline‐deficient diet‐induced NASH	EGCG	*Oxalobacter* ↓ *Oscillibacter* ↓ *Coprococcus_1* ↓ *Desulfovibrio* ↓ *norank_f__Bacteroidales_S24_7_group* ↑ *Alloprevotella* ↑ *Bacteroides* ↑	Long‐chain‐fatty‐acid‐CoA ligase ACSBG ↓ Lipid accumulation ↓	^167^
	Alcohol‐induced liver disease	Porphyromonas gingivalis	*Bacteroides* ↑ *Prevotella* ↑ *Desulfovibrio* ↑ *Lactobacillus* ↓	ACSL4 ↑ GPX4 ↓ SLC7A11 ↓ Ptgs2 ↑ Ncoa4 ↑ Mitochondrial function ↓ ATP ↓ Mitochondrial respiratory complexes ↓	^168^
	Acute ethanol‐induced liver injury	Ougan (OG), mulberry (MB), apple (AP), turnjujube (TJ)	*Dubosiella* ↑ *Lactobacillus* ↑ *Bifidobacterium* ↑ *Enterococcus* ↓ *Romboutsia* ↓ *Lachnospiraceae* ↓	NRF2 ↑ GPX4 ↑ ACSL4 ↓ GSH ↑ Iron overload ↓ ROS ↓	^169^
	APAP‐induced hepatotoxicity	Daidzein liberated by Lactobacillus vaginalis β‐Galactosidase	*Lactobacillus vaginalis* ↓ *Sutterella* ↑ *Bacteroides* ↑ *Lactobacillus* ↓	GPX4 ↑ xCT ↑ Fdps ↓ GSH ↑ ROS ↓ Lipid peroxidation ↓ AKT–GSK3β–Nrf2 axis ↑	^170^
	Hepatic I/R injury	Metformin	*Bacteroides* ↑ *Bacteroides thetaiotaomicron* ↑ *Bacteroides uniformis* ↑ *Bacteroides salyersiae* ↑	GABA ↑ ACSL4 ↓ TFR1 ↓ VDAC1,2,3 ↓ FTH1 ↑	^171^
	Palmitic acid‐induced hepatocyte injury	Bifidobacterium bifidum BGN4 fractions		MDA ↑ GSH ↓ SOD ↓ ROS ↑ Fe^2^⁺ ↑ GPX4 ↓ TFR1 ↑ SREBP1–CYP2E1 pathway ↓	^172^
	AFB1‐induced liver toxicity	Total flavonoids of rhizoma drynariae	*Firmicutes* ↓ *Proteobacteria* ↓ *Tenericutes* ↑ *Verrucomicrobia* ↑ *Lachnospiraceae* ↑ *Lactobacillaceae* ↑ *Lactobacillus* ↑ *[Ruminococcus]* ↑ *Faecalibacterium* ↓ *Subdoligranulum* ↓	Inhibition: GPX4 ↑ FTH1 ↑ ACSL4 ↓ GSH ↑ GSH/GSSG ratio ↑ Taurolithocholic acid ↓	^173^
	APAP‐induced acute liver injury	Oral fecal gavage, butyrate	*Lachnospiraceae* ↑ *Atopobiaceae* ↑ *Oscillospiraceae* ↑	AMPK–ULK1–p62 axis ↑ Mitophagy ↑ Nrf2 ↑	^174^
Cardiovascular disease	Diabetes mellitus and myocardial ischemia–reperfusion injury induced	DAPA	*Bacteroidetes* ↑ *Firmicutes* ↓ *Proteobacteria* ↑ *Escherichia‐Shigella* ↑ *Prevotella_9* ↑ *Muribaculaceae* ↓	TMAO ↑ in DIR group, ↓ with DAPA; ALB ↑ HMOX1 ↑ PPARG ↑ CBS ↑ LCN2 ↑ PPARA ↑	^192^
	Diabetes‐induced cardiomyopath	Salidroside	*Lactobacillus* ↓ *Bacteroidetes* ↑ *Firmicutes* ↓ *Enterobacter* ↓ *Bacteroides* ↑ *Alistipes* ↑	Iron overload ↓ GPX4 ↑ cTNT ↓ SLC7A11 ↓ LC3II ↓	^198^
	Doxorubicin‐induced cardiotoxicity	EMO	*Bacteroidota* ↑ *Verrucomicrobiota* ↓	Nrf2 pathway ↓	^200^
Other diseases	Pancreatic ductal adenocarcinoma	Clostridium butyricum, butyrate		Ferroptosis susceptibility ↑ Lipid peroxidation ↑ AMPK activation ↑	^201^
	CCl₄‐induced nephrotoxicity	Mori fructus aqueous extracts	*Akkermansia* ↑ *Anaerotruncus* ↑ *Clostridium_sensu_stricto* ↑ *Ihubacter* ↑ *Alcaligenes* ↑ *Dysosmobacter* ↑ *Clostridium_XlVa* ↓ *Helicobacter* ↓ *Paramuribaculum* ↓	Nrf2 signaling pathway ↑ KEAP1 ↓ HMGB1 ↓ ACSL4 ↓ TXNIP ↓ GPX4 ↑ HO‐1 ↑ SLC7A11 ↑ ZO‐1 ↑ Occludin ↑	^203^
	Benzene‐induced hematopoietic toxicity	Probiotics	*Bacteroidaceae* ↑	Fe^2^⁺ ↑ ROS ↑ lipid peroxidation ↑ ferroptosis protein ↑ IL‐5 ↑ IL‐13 ↑	^202^
	Ochratoxin A‐induced renal damag	Phytic acid	*Lactobacillus* ↑ *Escherichia‐Shigella* ↓ *Erysipelotrichaceae* ↓ *Clostridia_UCG‐014* ↓ *Ruminococcus_torques_group* ↓ *Faecalibacterium* ↑ *Firmicutes* ↑ *Proteobacteria* ↓	GPX4 ↓ SLC7A11 ↓ HO‐1 ↑ ACSL4 ↑ TfR ↑ MDA ↑	^204^
	Complete Freund's adjuvant‐induced pain and anxiety	Gastrodin	*Bacteroidetes* ↓ *Firmicutes* ↓	FTH1 ↓ GPX4 ↓ HO‐1 ↑ PTGS2 ↑	^205^
	T2DM mellitus‐associated cognitive dysfunction	Sinomenine	*Bacteroides* ↓ *Bifidobacteria* ↓ *Clostridia* ↓ *Enterobacteriaceae* ↓ *Lactobacillus* ↑ *Firmicutes* ↑ *Prevotella* ↑ *Megamonas* ↑ *Faebacterium* ↑	EGF ↑ Nrf2 ↑ HO‐1 ↑ ROS ↓ MDA ↓ Fe^2+^ ↓	^206^
	Mercury chloride‐induced brain injury	Mercury chloride	*Aeromonas hydrophila* ↑	MDA ↑ Fe^2+^ ↑ GSH ↓ ATP ↓ Mitochondrial damage ↑	^207^
	Ischemia–reperfusion‐induced cerebral ferroptosis	BBR	*Muribaculaceae* ↓ *Erysipelotrichaceae* ↓ *Helicobacteraceae* ↓ *Streptococcaceae* ↓ *Tannerellaceae* ↓ *Bacteroidaceae* ↑ *Enterobacteriaceae* ↑	GSH ↑ ROS ↓ GPX1 ↑ SLC7A11 ↑ ACSL4 ↓ Transferrin receptor 1 ↓	^209^
	Cigarette smoke extract and LPS‐induced lung injury	Polyphyllin B	*Akkermansia* ↑ *Escherichia‐Shigella* ↑ *Bacteroides* ↓ *Alloprevotella* ↓ *Parabacteroides* ↓ *Parasutterella* ↓	STAT3/NCOA4 pathway ↓ Ferritinophagy ↑ GPX4 ↑ Fe3+ deposition ↓ IL‐6 ↓ TNF‐α ↓ Oxidative stress ↓	^208^

### 5.4 Cell death in intestinal organ axis‐related diseases

#### 5.4.1 pyroptosis in liver diseases

In liver diseases, there is a significant connection between pyroptosis and the gut microbiota. Dysbiosis of the gut microbiota is considered a key factor in the development of various liver diseases, including NAFLD, alcoholic liver disease (ALD), autoimmune hepatitis, and cirrhosis. The gut microbiota directly affects liver health through the “gut–liver axis.” Pathogenic bacteria and harmful metabolites (such as endotoxins) from the gut enter the liver via the portal vein, triggering an inflammatory response that activates the NLRP3 inflammasome, subsequently inducing pyroptosis in hepatocytes. Pyroptosis primarily contributes to liver diseases by promoting inflammatory cell death in hepatocytes. This form of death releases large amounts of proinflammatory cytokines like IL‐1β and IL‐18, exacerbating liver inflammation and fibrosis, potentially leading to cirrhosis and liver cancer.^106^, ^107^

Disturbances in BA metabolism have a significant impact on the gut–liver axis, often leading to gut microbiota imbalance and liver inflammation. When BA homeostasis is disrupted, pathogenic bacteria such as Enterobacteriaceae overgrow, while beneficial bacteria like Akkermansia muciniphila decrease. This imbalance compromises the intestinal barrier, allowing bacterial components, such as LPS, to translocate through the gut into the liver via the portal vein. Once these substances enter the liver, they activate Kupffer cells (KCs) and hepatic stellate cells (HSCs) through the TLR4 receptor, triggering an inflammatory response and promoting liver fibrosis.^108^ The NLRP3 inflammasome, as an intracellular PRR, detects these pathogens or danger signals and mediates innate immune responses in the liver or gut. LPS binds to TLR4, initiating intracellular signaling pathways, with NF‐κB activation being a critical step. This process leads to the upregulation of proinflammatory cytokine precursors, such as IL‐1β and IL‐18, at the gene level. However, these precursor molecules must undergo further maturation via the NLRP3 inflammasome. The activation of the NLRP3 inflammasome depends on upstream TLR4 signaling, assembling a multiprotein complex with ASC and Caspase‐1, which induces pyroptosis and exacerbates the inflammatory response. Additionally, LPS–TLR4 signaling modulates metabolic pathways, such as glycolysis, and increases ROS levels, further enhancing NLRP3 activity and intensifying inflammation. In certain cases, LPS can directly enter cells and activate NLRP3 independently of TLR4, highlighting the complex interactions between these signaling pathways.^109^, ^110^ Specifically, the NLRP6 inflammasome plays a crucial role in maintaining gut microbiota balance by promoting IL‐18‐dependent antimicrobial peptide synthesis and goblet cell mucus secretion, thereby preserving the gut barrier and microbial equilibrium. In contrast, the NLRP3 inflammasome exacerbates inflammatory liver injury by inducing IL‐1β production. BAs, such as DCA and chenodeoxycholic acid, can activate the NLRP3 inflammasome in macrophages, further promoting inflammation. However, BAs may also exert the opposite effect by interacting with membrane‐bound Takeda G protein receptor 5 (TGR5) or activating the nuclear receptor FXR to inhibit NLRP3 inflammasome activity.^111^ The composition of the gut microbiota and its metabolites significantly influence NLRP3 inflammasome activation. For instance, when BA levels in the gut are reduced, dysbiosis occurs, particularly with an increase in Gram‐negative bacteria like *Bacteroidetes*. These bacteria and their LPS products can enter the liver through the portal vein, activating the hepatic NLRP3 inflammasome and promoting IL‐1β production, which intensifies liver inflammation. Moreover, the NLRP3 inflammasome plays a dual role in regulating gut barrier function. Under normal conditions, it maintains microbial balance and protects the intestinal mucosa by promoting IL‐18 synthesis. However, when the gut barrier is damaged, gut microbes and their antigens may penetrate the intestinal lamina propria. At this level, pathogen‐associated molecular patterns can be recognized by macrophages and dendritic cells via PRR, leading to NLRP3 inflammasome activation in macrophages, inducing IL‐1β production, and exacerbating inflammation.^112^, ^113^

In autoimmune hepatitis, prebiotic interventions regulate gut microbiota by increasing populations such as *Rikenella* and *Alistipes* while reducing *Escherichia‐Shigella* and *Lactobacillus*. This intervention also inhibits the activation of the NLRP3 inflammasome and the TLR4/NF‐κB signaling pathway.^114^ Liver injury induced by polyethylene microplastics is characterized by an increase in bacterial populations such as *Desulfovibrio* and *Clostridia*, accompanied by the activation of the TLR2/NF‐κB/NLRP3 signaling pathway.^115^ In the treatment of NAFLD, the herbal formula Xie Zhuo Tiaozhi Tang increases beneficial bacteria such as *Ileibacterium valens* and *A. muciniphila*, while reducing the expression of inflammatory cytokines and pyroptosis‐related proteins.^116^ In alcohol‐induced liver diseases, liver injury caused by huangjiu (yellow rice wine) is exacerbated by histamine, which leads to alterations in gut microbiota, such as an increase in *Prevotellaceae UCG‐001*, and activates pyroptosis pathways.^117^ Compound probiotics show protective effects in alcohol‐induced liver injury by modulating gut microbiota and inhibiting the TLR4/NF‐κB signaling pathway.^118^ Butyrate mitigates alcoholic fatty liver by reducing *Parabacteroides* populations and inhibiting GSDMD‐mediated pyroptosis.^119^ In addition, lycopene demonstrates protective effects in high‐fat, high‐fructose diet‐induced liver injury by decreasing *Firmicutes* and *Lachnospiraceae* populations and downregulating the NLRP3 inflammasome signaling pathway.^120^ AFB1 exposure not only increases *Bacteroides* populations but also decreases *Alistipes* and upregulates the TLR4/NLRP3/caspase‐1 signaling pathway, aggravating liver pyroptosis. AFB1 also elevates levels of pipecolic acid and norepinephrine.^121^, ^122^ Ginsenoside Rg2 alleviates aging‐related liver damage by promoting the growth of *Firmicutes* and inhibiting the NLRP3 inflammasome pathway.^123^ Paeoniflorin protects against zinc oxide nanoparticle‐induced hepatotoxicity by modulating gut microbiota and inhibiting the NLRP3 inflammasome.^124^ In tetrachlorodibenzo‐p‐dioxin‐induced hepatotoxicity, significant changes in gut microbiota were observed, including a decrease in *Firmicutes* and an increase in *Nitrospirota*, accompanied by mitochondrial quality control imbalance and increased necroptosis.^125^ Peptides derived from corn protein alleviate high‐fat diet‐induced liver fibrosis by modulating gut microbiota and inhibiting the NLRP3 inflammasome.^126^ In methionine‐choline‐deficient high‐fat diet‐induced nonalcoholic steatohepatitis (NASH), paeoniflorin reduces inflammation by inhibiting the NLRP3 inflammasome and activating the AhR pathway.^127^ Geraniol enhances intestinal barrier function in bisphenol A‐induced liver injury by modulating gut microbiota and inhibiting the NLRP3/caspase‐1 signaling pathway.^128^ In NASH induced by a high‐fat, high‐cholesterol diet combined with nicotine, *Bacteroides xylanisolvens* promotes NASH progression by affecting the AMPKα signaling pathway and nicotine accumulation.^129^ Diallyl disulfide mitigates ethanol‐induced hepatic steatosis and inflammation by increasing populations of *Rikenella* and *Alistipes* and inhibiting the NLRP3 and NF‐κB signaling pathways.^130^ Splenectomy reduces inflammation in concanavalin A‐induced cirrhosis by decreasing *Veillonella* populations and inhibiting the TLR4/NLRP3 signaling pathway (Table 1).^131^

#### 5.4.2 pyroptosis in cerebral disease

Gut microbiota‐derived metabolites, including SCFAs (such as butyrate and acetate), TMAO, indole derivatives, and secondary BAs, regulate host immune responses through various pathways and play a crucial role in the activation of the NLRP3 inflammasome. The gut microbiota and the NLRP3 inflammasome play crucial roles in regulating brain function, as evidenced by studies on germ‐free (GF) mice and antibiotic‐treated mice. The gut microbiota influences various biological processes in the CNS, including development, neurogenesis, neurotransmission, immune activity, and maintenance of the BBB.^132^, ^133^ Following antibiotic treatment, the mRNA expression of caspase‐1, ASC, IL‐1β, and IL‐18 was significantly upregulated in the gut and brain tissues of mice, suggesting that the gut microbiota plays a key role in regulating the expression of inflammasome components.^134^ The importance of the NLRP3 inflammasome in the GBA was further demonstrated in mouse models, where NLRP3 knockdown led to changes in gut microbiota composition and behavior. Additionally, mice lacking caspase‐1 exhibited differences in both gut microbiota composition and behavior compared with wild‐type (WT) mice.^135^ In the pathogenesis of neurodegenerative diseases and psychiatric disorders, several key mechanisms are involved. First, the NLRP3 inflammasome triggers neuroinflammation through the activation of IL‐1β and IL‐18. Second, the aggregation of Aβ and α‐syn accelerates the formation of the NLRP3 inflammasome, leading to pyroptosis and further exacerbating neurodegeneration. Additionally, changes in the gut microbiota regulate NLRP3 activation, affecting the homeostasis of the GBA. Oxidative stress‐related stimuli, such as ROS, can also activate NLRP3, further intensifying neuroinflammation and mitochondrial dysfunction. Last, microbial‐derived metabolites, such as indole and TMAO, either inhibit or activate the NLRP3 inflammasome, influencing neuroinflammation and cognitive dysfunction. These changes in key proteins form a complex connection between the gut microbiota, NLRP3 inflammasome‐mediated pyroptosis, and CNS diseases.^2^

Different intervention factors significantly influence the composition of the gut microbiota and its interaction with the NLRP3 inflammasome signaling pathway, playing a crucial role in CNS diseases. For example, Bazi Bushen capsules significantly increase the abundance of *Firmicutes* and related bacteria in aging‐induced gut barrier dysfunction, while inhibiting the NLRP3 inflammasome signaling pathway and reducing the expression of proinflammatory factors such as IL‐1β, IL‐18, and GSDMD, potentially exerting anti‐inflammatory effects.^136^ Temperature stress experiments showed that under low‐temperature conditions, the abundance of *Bacteroidetes* and *Verrucomicrobia* increased, and the expression of the NLRP3 inflammasome and downstream inflammatory factors decreased. Conversely, high‐temperature conditions promoted NLRP3 inflammasome activation and elevated levels of proinflammatory factors like IL‐1β and TNF‐α.^137^ In a LPS‐induced sepsis‐associated encephalopathy model, the NU9056 treatment group exhibited an increase in *Verrucomicrobia* and *Akkermansia*, along with a significant reduction in NLRP3 inflammasome activity, alleviating the inflammatory response.^138^ In the 5xFAD mouse model of AD, significant changes in gut microbiota were observed, including a decrease in *Firmicutes* and an increase in *Bacteroidetes*, along with overactivation of the NLRP3 inflammasome, suggesting that dysbiosis may play a key role in the occurrence and progression of neuroinflammation.^139^ Moreover, environmental stressors such as long‐term unpredictable mild stress and chronic sleep deprivation also activate the NLRP3 inflammasome by altering the gut microbiota composition (e.g., reducing *Lactobacillaceae*), leading to neuroinflammation and pyroptosis. This highlights the critical influence of gut microbiota‐NLRP3 inflammasome interactions in CNS diseases.^140^, ^141^, ^142^, ^143^, ^144^ These studies emphasize the potential therapeutic role of modulating gut microbiota and the NLRP3 inflammasome signaling pathway in treating CNS disorders (Table 1).

#### 5.4.3 pyroptosis in CVDs

In the development and progression of atrial fibrillation (AF) and AS, gut microbiota metabolites play a critical role by activating the NLRP3 inflammasome. When the balance of a healthy gut microbiota is disrupted, significant changes occur in metabolites such as SCFAs, BAs, TMAO, and indoxyl sulfate. These metabolites are directly or indirectly involved in the activation of the NLRP3 inflammasome, thereby exacerbating the pathological processes of AF and AS. SCFAs are essential for maintaining gut homeostasis and host immunity. They activate the NLRP3 inflammasome through G protein‐coupled receptors and help repair the gut barrier, reducing the infiltration of harmful substances. In AF patients, SCFA levels are reduced, but supplementation of SCFAs has been shown to decrease atrial fibrosis and NLRP3 inflammasome activation. BAs, as endogenous ligands, activate the NLRP3 inflammasome through calcium influx pathways and regulate its activity via the FXR. TMAO promotes NLRP3 inflammasome activation by inhibiting the sirtuin 3 (SIRT3)–superoxide dismutase 2 (SOD2)–mitochondrial ROS signaling pathway, leading to cardiovascular inflammation and worsening the pathological progression of AF and AS. IS contributes to AF risk by upregulating key components of the NLRP3 inflammasome, leading to myocardial pyroptosis and fibrosis.^145^ In AS patients, gut dysbiosis, characterized by an increase in *Firmicutes* and a decrease in *Bacteroidetes*, is closely related to lipid metabolism disorders and enhanced inflammation. The NLRP3 inflammasome promotes AS progression through the NF‐κB signaling pathway and the ROS–thioredoxin‐interacting protein (TXNIP)–NLRP3 pathway, while cholesterol crystal deposition further activates the inflammasome, triggering plaque inflammation. Therefore, gut microbiota metabolites significantly influence the occurrence and progression of AF and AS by activating the NLRP3 inflammasome.^146^

In a study on age‐related AF in elderly rats and humans, fecal microbiota transplantation led to significant changes in the gut microbiota, including a reduction in *Firmicutes*, an increase in *Bacteroidetes* and *Spirochaetae*, and a decrease in *Verrucomicrobia*. Additionally, there was a reduction in *Lactobacillus* spp. and an increase in *Bacteroidaceae*. These changes were accompanied by the activation of the NLRP3 inflammasome, the TLR4/NF‐κB pathway, elevated LPS levels, increased glucose, atrial fibrosis, and elevated levels of IL‐1β, transforming growth factor β (TGF‐β1), and alpha‐smooth muscle actin (α‐SMA).^147^ In another study on cold exposure‐induced AF, changes in the gut microbiota associated with *A. muciniphila* were found to elevate TMAO, caspase‐1, cleaved GSDMD, TGF‐β1, and α‐SMA levels.^148^ Furthermore, in a study on aortic dissection induced by angiotensin II and β‐aminopropionitrile fumarate, increases in gut microbiota related to *Escherichia‐Shigella*, *Alistipes*, *Parabacteroides*, *Klebsiella*, and *Akkermansia* were observed, along with upregulation of NLRP3, ASC, caspase‐1, and TLR4 (Table 1).^149^

#### 5.4.4 pyroptosis in other diseases

In a cecal ligation and puncture (CLP)‐induced sepsis model, treatment with “Qi Huang Fang” increased the abundance of *Firmicutes*, *Muribaculaceae*, *Campilobacterota*, *Helicobacter*, *Alistipes*, *Bacteroidetes*, and *Actinobacteria*, while inhibiting GSDMD‐mediated pyroptosis and the NLRP3 inflammasome pathway. It also upregulated the expression of tight junction proteins (Zonula occludens‐1 (ZO‐1), Occludin).^150^ In another CLP‐induced sepsis model, fecal microbiota transplantation and SCFA treatment led to a reduction in *Firmicutes* and an increase in *Proteobacteria* and *Escherichia‐Shigella*, accompanied by downregulation of NLRP3, GSDMD‐N, and IL‐1β.^151^ In a pregnancy‐induced sepsis model, *Parabacteroides merdae* promoted macrophage pyroptosis by upregulating the Flavin mononucleotide (FMN)–Heterogeneous nuclear ribonucleoprotein U‐like 2 (hnRNPUL2)–NLRP3 signaling axis.^152^ In AFB1‐induced spleen injury, the *Bacteroidota/Firmicutes* ratio decreased, along with an increase in *Firmicutes* and *Lactobacillus*. This was accompanied by upregulation of LPS, the TLR4/NF‐κB/NLRP3 signaling pathway, and pyroptosis markers (IL‐1β, IL‐18, Caspase‐1, GSDMD.^153^ In dehydroepiandrosterone (DHEA)‐induced PCOS, treatment with disulfiram and metformin resulted in a decrease in *Akkermansia* and an increase in *Desulfovibrio* and *Burkholderia*, along with reductions in GSDMD, Caspase‐1, LPS, and IFN‐γ levels. Estrogen synthesis was inhibited, and granulosa cell apoptosis increased.^154^ In high‐fat diet‐induced metabolic endotoxemia, *Proteobacteria* increased, while the *Firmicutes/Bacteroidota* ratio decreased. This was accompanied by an increase in GSDMD‐N/CL binding, along with upregulation of Caspase‐1, IL‐1β, and LPS. Gut permeability was also compromised, with decreased expression of ZO‐1, Occludin, and Claudin (Table 1).^155^

#### 5.4.5 Ferroptosis in liver disease

In the context of liver diseases, the regulation of iron homeostasis is particularly crucial. Both in humans and animals, a strict regulatory mechanism exists for maintaining iron balance, with the liver serving as the primary site for iron storage and regulation through the hormone hepcidin.^156^ Hepcidin, produced in hepatocytes, plays a key role in iron metabolism.^157^ However, iron overload can lead to liver damage, contributing to conditions such as liver fibrosis and liver cancer.^158^ Therefore, in liver diseases, hepcidin is closely related to iron metabolic disorders and programmed cell death, specifically ferroptosis. For instance, in ALD, patients exhibit diminished serum hepcidin levels, while the expression of the divalent metal ion transporter in the gut increases. This results in elevated serum iron and FTH1 levels, which are linked to the key characteristics of ferroptosis, highlighting the important role of hepcidin in both iron metabolic disorders and ferroptosis in liver diseases.^159^ Recent studies have shown that ferroptosis plays a key role in the development of NASH and the progression of NAFLD. Gut microbiota, through the gut–liver axis and its metabolites, such as SCFAs, BAs, and endogenous ethanol, influence the process of ferroptosis. BAs regulate liver metabolism and inflammation via the FXR and G protein‐coupled BA receptor, and dysbiosis of the gut microbiota may exacerbate ferroptosis by modulating these pathways. SCFAs reduce ferroptosis by maintaining gut barrier function and regulating lipid metabolism, while increased endogenous ethanol can enhance gut permeability, promote liver inflammation, and lipid peroxidation, thus triggering ferroptosis.^160^

In nonalcoholic liver injury, a Western diet (rich in fats, sugars, and cholesterol)‐induced NASH, which was accompanied by an increase in *Bacteroides*, *Rikenellaceae*, and *Helicobacteraceae*, and a decrease in *Lachnospiraceae*, *Desulfobacterota*, and *Firmicutes*. These changes were associated with increased lipid peroxidation, downregulation of GPX4 and SLC7A11, and eventual liver fibrosis, with markers TGF‐β1 and collagen type I alpha 1 (COL1A1) upregulated.^161^ In bromopyruvate‐induced metabolic‐associated fatty liver disease (MAFLD), upregulation of the TFR–ACSL4 signaling pathway, decreased GSH levels, dysregulated iron metabolism, and elevated ROS were observed.^162^ In nonalcoholic fatty liver induced by *Porphyromonas gingivalis* infection, *Bacteroidetes*, *Helicobacter*, and *Prevotella* increased, while *Coprococcus*, *Desulfovibrio*, and *Firmicutes* decreased. GPX4 and SLC7A11 were downregulated, ACSL4 was upregulated, and Th17/Treg imbalance occurred.^163^ After nobiletin treatment, *Ligilactobacillus*, *Akkermansia*, and *Lactobacillus* increased, while *Dubosiella* and *Bacteroides* decreased, enhancing the Nrf2–GPX4 signaling pathway, elevating GSH levels, and reducing ROS and iron metabolism.^164^ In a high‐fat diet‐induced fatty liver transplant model, Ferrostatin‐1 (Fer‐1) treatment promoted an increase in *Peptostreptococcaceae*, *Muribaculaceae*, and *Prevotellaceae*, while inhibiting *Enterobacteriaceae* and *Fusobacteriaceae*, and upregulated GPX4, reduced lipid ROS, and regulated iron metabolism.^165^ In a choline‐deficient, high‐fat diet (CDAHFD)‐induced NAFLD model, urolithin C increased *Parabacteroides goldsteinii* and *Lactobacillus vaginalis*, inhibited several bacteria such as *Helicobacter ganmani*, and increased MUFAs through the AMPK signaling pathway, reducing lipid peroxidation.^166^ Similarly, in an MCD diet‐induced NASH model, treatment with epigallocatechin gallate (EGCG) led to significant changes in the gut microbiota, reducing *Oxalobacter*, *Oscillibacter*, *Coprococcus_1*, and *Desulfovibrio*, while increasing unidentified *Bacteroidales S24_7 group* strains, *Alloprevotella*, and *Bacteroides*. EGCG significantly inhibited the expression of ACSBG, reducing lipid accumulation.^167^

In alcohol‐induced liver injury, *P. gingivalis* infection caused increases in *Bacteroides*, *Prevotella*, and *Desulfovibrio*, along with a decrease in *Lactobacillus*. This was accompanied by upregulation of ACSL4, downregulation of GPX4 and SLC7A11, mitochondrial dysfunction, and reduced ATP production.^168^ In acute alcohol‐induced liver injury, treatments with Ougan, mulberry, apple, and Turnjujube increased *Dubosiella*, *Lactobacillus*, and *Bifidobacterium*, reduced iron overload and lipid peroxidation, and improved oxidative stress by enhancing NRF2 and GPX4 expression.^169^

In other liver injuries, acetaminophen (APAP)‐induced drug‐induced liver toxicity was regulated by β‐galactosidase released from *Lactobacillus*, which modulated GPX4 and GSH, inhibiting lipid peroxidation and alleviating oxidative stress through the AKT–glycogen synthase kinase 3 beta (GSK3β)–Nrf2 axis.^170^ ischemia–reperfusion‐induced liver injury, metformin increased the abundance of *Bacteroides* and its subspecies, such as *Bacteroides thetaiotaomicron*, regulated GABA levels, inhibited ACSL4 and transferrin receptor 1 (TFR1) expression, and restored iron metabolism by promoting FTH1.^171^ Palmitic acid‐induced hepatocyte injury showed elevated MDA and ROS levels, along with downregulation of GPX4 and GSH, and increased iron overload.^172^ In AFB1‐induced hepatotoxicity, total flavonoids reduced hepatic lipid peroxidation by inhibiting ACSL4 and enhancing GPX4 and FTH1 expression.^173^ In APAP‐induced acute liver injury, fecal microbiota transplantation with oral butyrate increased *Lachnospiraceae* and *Atopobiaceae* and promoted mitophagy by activating the AMPK–Unc‐51‐like autophagy activating kinase 1 (ULK1)–sequestosome 1 (p62) axis and Nrf2 signaling pathway (Table 2).^174^

#### 5.4.6 Ferroptosis in CVD

Ferroptosis is closely associated with the onset and progression of various cardiac conditions, including myocardial I/R damage, heart failure, cardiomyopathy, and AS. In these diseases, ferroptosis may be linked to lipid accumulation, iron metabolism disorders, and increased ROS levels in vascular smooth muscle cells, endothelial cells (ECs), and macrophages. The application of drugs or genetic modification techniques to specifically inhibit ferroptosis has shown promising therapeutic effects.^175^, ^176^ For example, mice lacking the cardiac transferrin receptor, Tfr1, in the heart experience severe cardiomyopathy and cardiac iron deficiency.^177^ Similarly, cardiomyocyte‐specific Fth knockout mice display cardiac hypertrophy and severe myocardial injury. In a genetic mouse model with heart‐specific conditional knockout of FTH1, overexpression of Slc7a11 effectively reverses cardiomyopathy caused by FTH1 deficiency by blocking ferroptosis in cardiomyocytes.^178^

In the early stages of heart failure, circulating hepcidin levels increase, though this is not accompanied by anemia or inflammation. However, as heart failure progresses, circulating hepcidin levels decrease, which is related to iron deficiency. This fluctuation in hepcidin levels can influence ferroptosis, as changes in iron homeostasis impact the susceptibility of cells to iron‐dependent oxidative damage.^179^

The “gut–heart axis” plays a crucial role in CVD research, with the gut microbiota significantly influencing ferroptosis in cardiac conditions. For instance, in heart failure patients, gut microbiota dysregulation leads to a reduction in beneficial bacteria, such as those producing SCFAs. Iron deficiency in the host results in a decrease in butyrate‐producing bacteria in the gut microbiota, and the consequent decrease in butyrate synthesis can increase the risk of heart failure and anxiety.^180^, ^181^ Additionally, the metabolism of phospholipids in the gut microbiota promotes the development of CVDs by converting choline, phosphatidylcholine, and phosphatidylethanolamine into trimethylamine, which is subsequently oxidized in the liver to TMAO, a compound linked to increased cardiovascular risk.^182^

Ferroptosis influences AS through various signaling pathways, including alterations in lipid profiles, formation of atherosclerotic and fibrous plaques, and plaque rupture. It is also associated with inflammation, exacerbating the poor prognosis of AS‐related diseases.^183^ Specifically, ferroptosis in vascular ECs (VECs) can exacerbate AS by promoting endothelial dysfunction. It triggers the early stages of AS by increasing endothelial damage and death, caused by the disruption of cellular and mitochondrial membranes. Concurrently, ferroptosis in VECs stimulates plaque angiogenesis, suggesting that vascular remodeling induced by VEC ferroptosis is crucial for the stabilization of atherosclerotic plaques in the intermediate to late stages of the disease.^184^ Metagenomic sequencing of fecal microbiota has shown differences in microbial composition between patients with unstable plaques and those with stable plaques. Specifically, there is an increase in lactic acid bacteria and a decrease in *Roseburia*. The presence of unstable plaques correlates with a reduced abundance of *Roseburia* in fecal samples. Concurrently, the microbiota's capacity to produce proinflammatory peptidoglycans has increased, while the synthesis of anti‐inflammatory carotenes has decreased. Therefore, the gut microbiota of CVD patients may promote inflammation by producing proinflammatory molecules.^185^ Moreover, a study revealed that Qing‐Xin‐Jie‐Yu Granule inhibits ferroptosis in fragile atherosclerotic plaques and reshapes the gut microbiota.^186^

Oxidative stress is a pivotal factor in I/R injury and acute myocardial infarction.^187^ During ischemia and the initial stages of reperfusion, FTH1 breakdown releases iron, driving the iron‐dependent Fenton reaction, which results in oxidative damage and a subsequent decline in cardiac function associated with I/R injury.^188^ Suppression of glutaminase metabolism, a key step in ferroptosis, has been shown to mitigate cardiac damage induced by I/R, further highlighting the role of ferroptosis in this context.^189^ Additionally, I/R injury in mice leads to significant intestinal mucosal damage and disruption of tight junctions, accompanied by rapid onset dysbiosis, characterized by an overgrowth of *Proteobacteria* and *Enterobacteriaceae*.^190^ The expansion of Proteobacteria disrupts gut microbiota balance, triggering mucosal inflammation and compromising gut barrier integrity. Interestingly, preliminary clearance of gut microbiota has been observed to substantially protect against myocardial I/R damage.^191^ In myocardial I/R injury, the use of dapagliflozin (DAPA) has been associated with changes in the gut microbiota, including an increase in *Bacteroidetes*, *Proteobacteria*, and *Escherichia‐Shigella*, and a decrease in *Firmicutes* and *Muribaculaceae*. These changes are related to higher TMAO levels in the diabetic ischemia–reperfusion (DIR) group. However, DAPA treatment reduced TMAO levels while increasing the expression of HMOX1, PPARG, CBS, LCN2, and PPARA.^192^

In parallel, adipokines such as leptin and adiponectin are critical in regulating CVDs. In conditions such as obesity, leptin can induce oxidative stress, inflammation in immune cells, and vascular smooth muscle hypertrophy.^193^ Oxidative stress initiation and substantial ROS generation can trigger cellular ferroptosis. Notably, administration of broad‐spectrum antibiotics in rats has been shown to affect leptin concentrations and the metabolites produced during the breakdown of aromatic amino acids, thereby reducing myocardial infarction severity.^194^, ^195^ Moreover, probiotics such as *Lactobacillus plantarum* and *Bifidobacterium lactis* can lower blood leptin levels by altering the gut microbiota in myocardial infarction models, improving cardiac ischemic tolerance, and reducing acute injury, hypertrophy, and cardiac remodeling after I/R.^194^ This suggests that gut microbiota modulates myocardial I/R damage by influencing ferroptosis via metabolites. For instance, ellagitannins—polyphenolic compounds found in pomegranates, walnuts, and berries—are metabolized by the gut microbiota into ellagic acid, which is subsequently hydrolyzed into biologically potent metabolites such as urolithin A and urolithin B (UB).^196^ These metabolites exhibit strong antioxidant and anti‐inflammatory properties. Research indicates that UB can regulate the p62/kelch‐like ECH‐associated protein 1 (Keap1)/Nrf2 signaling pathway, reducing overall oxidative stress and potentially improving outcomes in myocardial I/R injury.^197^ In addition, in diabetes‐induced cardiomyopathy, salidroside has been shown to regulate the gut microbiota by reducing *Lactobacillus* and *Enterobacter* populations while increasing *Bacteroidetes*, *Bacteroides*, and *Alistipes*. These changes are associated with reduced iron overload, increased expression of GPX4 and cardiac troponin T (cTNT), and decreased expression of SLC7A11 and microtubule‐associated protein 1 light chain 3 II (LC3II).^198^

Similarly, doxorubicin (DOX)‐induced cardiomyopathy has been closely linked to mitochondrial‐dependent ferroptosis. DOX decreases GPX4 expression and promotes lipid peroxidation in mitochondria via the DOX–Fe^2+^ complex, leading to mitochondrial‐dependent ferroptosis. This is evidenced by significant impairment in the left ventricular ejection fraction, increased cardiac fibrosis, and a rise in TUNEL‐positive cells in disseminated intravascular coagulation (DIC) mouse models.^199^ Cardiac injuries are reduced in GPX4 transgenic mice and exacerbated in GPX4 heterozygous knockout mice, underscoring the protective role of GPX4 against ferroptosis. In models of DOX‐induced cardiac toxicity, the increased expression of heme oxygenase‐1 (HO‐1), regulated by Nrf2, facilitates the release of free iron, triggering ferroptosis. Notably, the application of Fer‐1 significantly mitigates DOX‐induced DIC in mouse models. Additionally, combining Fer‐1 with the iron‐binding agent deferasirox notably enhances survival rates, suggesting an effective therapeutic strategy for reducing DOX‐induced cardiac damage. Alterations in gut microbiota composition also impact iron and lipid metabolism, thereby regulating ferroptosis and improving DIC outcomes. For example, Emodin (EMO) has been shown to regulate gut microbiota and influence ferroptosis in DIC. However, the protective effects of EMO are lost when the gut microbiota is removed by antibiotic treatment. EMO restores gut microbiota composition, disrupted by DOX, to near‐normal levels—an effect absent in Nrf2−/− mice, indicating that EMO's amelioration of DIC is tied to Nrf2‐dependent ferroptosis inhibition. Further analysis of serum metabolites reinforces the connection between gut microbiota and cardiac function (Table 2).^200^

#### 5.4.7 Ferroptosis in other diseases

In various disease types, the gut microbiota is closely linked to the ferroptosis pathway and plays a crucial role in different pathological processes. In cancer, a pancreatic ductal adenocarcinoma model showed that *Clostridium butyricum* and its metabolite butyrate significantly increased ferroptosis susceptibility, accompanied by lipid peroxidation and AMPK activation.^201^ In a benzene‐induced hematotoxicity model, probiotics regulated changes in gut microbiota such as *Bacteroidaceae*, leading to increased levels of iron (Fe^2^⁺) and lipid peroxidation, further inducing ferroptosis and upregulation of proinflammatory cytokines like IL‐5 and IL‐13.^202^ In kidney injury, a CCl₄‐induced nephrotoxicity model demonstrated that *Mori fructus* aqueous extract increased the abundance of *Akkermansia* and reduced ferroptosis‐related markers ACSL4 and TXNIP, while activating the Nrf2 signaling pathway, indicating the important role of gut microbiota in alleviating kidney damage.^203^ Similarly, in aspergillin A‐induced kidney injury, phytic acid modulated the gut microbiota (increasing *Lactobacillus* and decreasing *Escherichia‐Shigella*) and inhibited the expression of GPX4 and SLC7A11, leading to an increase in lipid peroxidation product MDA.^204^ In neurological diseases, the gut microbiota is also closely associated with ferroptosis. In a complete Freund's adjuvant‐induced pain and anxiety model, gastrodin reduced the abundance of *Bacteroidetes* and *Firmicutes*, promoting the reduction of FTH1 and increasing the expression of ferroptosis‐related markers like GPX4.^205^ Additionally, in T2DM‐related cognitive dysfunction, sinomenine regulated gut microbiota (increasing *Lactobacillus* and *Firmicutes*), reducing ROS and Fe^2^⁺ levels, and inhibited oxidative stress and ferroptosis by activating the Nrf2 pathway.^206^ In mercury chloride‐induced brain injury, the abundance of *Aeromonas hydrophila* in the gut increased, along with elevated levels of iron and MDA, leading to mitochondrial damage and ferroptosis.^207^ In lung injury, a model of cigarette smoke extract and LPS‐induced lung damage demonstrated that polyphyllin B increased the abundance of *Akkermansia* and *Escherichia‐Shigella*, inhibiting ferritinophagy and oxidative stress, while reducing iron (Fe^3^⁺) deposition and the expression of proinflammatory factors such as IL‐6 and TNF‐α.^208^ Moreover, in ischemia–reperfusion‐induced brain injury, berberine (BBR) reduced the abundance of *Muribaculaceae* and *Erysipelotrichaceae*, increased GSH levels, and inhibited the expression of ferroptosis‐related protein ACSL4, thereby mitigating brain damage (Table 2).^209^

## 6 ORGAN AND ORGANOID CHIP

The gut microbiota influences distant organs through the “gut–organ axis,” a mechanism that involves interactions across multiple physiological systems. Gut microbes regulate brain function via neural and endocrine pathways, such as through the GBA, and influence systemic glucose metabolism by modulating the secretion of glucagon‐like peptide‐1. Additionally, metabolites produced by gut microbes, such as SCFAs, can impact distant organs like the liver and bones by modulating the immune system. Moreover, the metabolic products of the gut microbiota can directly participate in systemic metabolic regulation through the circulatory system, further affecting the health of distant organs.^210^, ^211^, ^212^

It is clear that to precisely validate how the gut microbiota influences cell death in disease, one cannot rely solely on observing changes in microbiota composition or cell death markers, nor can this be achieved indirectly through in vitro drug intervention experiments. In vitro experiments typically occur in two‐dimensional (2D) cultures, which fail to fully simulate the three‐dimensional (3D) structure and complex microenvironment of the gut, including cell interactions and microbial diversity. However, studying how the gut microbiota specifically influences cell death in diseases through in vivo experiments is highly challenging. The complexity of the gut microenvironment and microbiota in vivo is difficult to control, leading to poor reproducibility and interpretation of experimental results. Isolating the independent effects of gut microbiota, the immune system, and other physiological factors on cell death is difficult. Therefore, systematically studying how drug metabolites and gut microbial metabolites influence cell death through the gut–organ axis is crucial.^213^, ^214^

**FIGURE 3:**
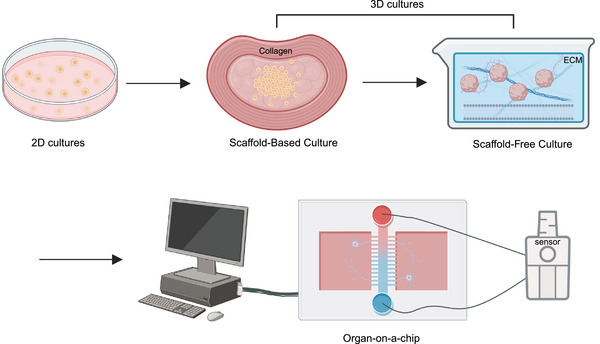
Progression from 2D cultures to organ‐on‐a‐chip models. The evolution of cell culture techniques, starting from traditional 2D cultures where cells are grown on flat surfaces. The progression moves to 3D cultures, which can either be scaffold‐based, where cells are supported by materials like collagen, or scaffold‐free, where cells self‐organize in a matrix such as the ECM. The final stage represents the development of organ‐on‐a‐chip technology, where cells are cultured in microfluidic devices that mimic organ functions.

Organ‐on‐a‐chip technology offers a controlled and highly physiologically relevant environment to systematically study how the gut microbiota and its metabolites affect cell death. This approach not only overcomes the limitations of traditional in vitro and in vivo experiments but also provides more precise and in‐depth mechanistic insights. The development of cell culture methods has evolved from simple 2D monolayer cultures to more sophisticated 3D culture techniques. 2D cell culture, while widely used and critical for pharmacokinetics research, remains the foundation of drug safety and toxicity studies. However, 2D cultures cannot simulate hypoxic conditions and metabolic zoning, which may affect cell proliferation behavior in cancer pathology and certain tissue studies. In contrast, 3D cell culture techniques better simulate the complexity of in vivo tissues. These methods are categorized into scaffold‐based and scaffold‐free techniques. Scaffold‐based methods use natural materials to mimic the extracellular matrix (ECM), promoting cell attachment and interaction. Scaffold‐free methods, such as spheroid and organoid cultures, allow researchers to control concentration gradients of compounds that influence cell behavior, such as signaling, migration, and gene expression. Spheroid cultures create cell aggregates through techniques like hanging drop plates, microwell plates, magnetic levitation, microfluidic devices, and bioprinting, which better replicate in vivo tissue characteristics such as hypoxia, cell dormancy, and activation of cell death inhibition pathways. By utilizing enriched stem cells, induced pluripotent stem cells (iPSCs), or embryonic stem cells (ESCs) within a specific 3D culture environment, it is possible to simulate the structure and function of human organs. During cultivation, ECM components (e.g., collagen, laminin) and signaling molecules (e.g., wingless‐type MMTV integration site family, member 3A (WNT3A), R‐spondin) synergistically promote the spontaneous aggregation and differentiation of stem cells into organoids. Organoids exhibit diverse morphologies that vary depending on the cell source and the target organ. Spherical/cyst‐like organoids are composed of a single layer of epithelial cells and have a relatively simple structure, making them commonly used to model basic functions of organs such as the intestine and kidney. Branching organoids, which form complex branched structures during differentiation, are typically employed to mimic organs with branched features, such as the lung and mammary gland, effectively recapitulating the architecture of these tissues. Budding organoids undergo a budding process, forming more complex structures, and are often used to model multilayered developmental states in organs like the stomach and intestine. These organoids not only recapitulate complex in vivo organ functions, such as cell‐cell communication and gene regulation, but also provide a valuable platform for disease modeling and drug screening (Figure 3).^215^, ^216^, ^217^, ^218^

Organ‐on‐a‐chip is a highly engineered microfluidic device composed of microfluidic systems, cell culture matrices, biosensors, multicellular cocultures, hydrodynamic control, mechanical stimulation, and dynamic environment control systems. The microfluidic system uses a series of channels and chambers to precisely control fluid flow. Cells are typically cultured on matrix materials and monitored in real‐time through sensors. Organ chips simulate the physical and biochemical functions of human organs through precise hydrodynamics and mechanical stimulation, combined with dynamic environmental control. This highly controllable experimental platform is widely used in drug screening and disease research.^219^

Organoid‐on‐a‐chip technology is an advanced in vitro model that combines organoids with microfluidic systems to better simulate the structure and function of human organs. Organoids are 3D cell cultures derived from stem cells that can self‐organize and mimic the architecture of real organs. They can be developed from iPSCs or stem cells from primary human biopsy samples, providing greater cellular diversity than traditional models, such as immortalized Caco‐2 cell lines. However, organoids lack key elements of the in vivo gut microenvironment, such as the vascular system and mechanical forces from fluid flow and peristalsis, which limits their physiological relevance. Additionally, the spherical structure of organoids presents experimental challenges, including inconsistencies in size and shape, poor control over critical variables, and limited access to only one side of the epithelium.^220^, ^221^ In recent years, organ‐on‐a‐chip technology has made significant advances, particularly in spatial‐temporal signal control, mechanical cue modeling, high‐throughput analysis, coculture and multitissue interactions, as well as the integration of biosensing and bioimaging. By regulating spatial concentration, temporal signal combinations, and morphology guidance, these systems precisely mimic the complex physiological microenvironments in vivo. In terms of mechanical cue modeling, the chips can apply shear forces, periodic compression, and stretching to simulate the mechanical stress present in the body. High‐throughput analysis techniques, such as microwell arrays, droplets, and acoustic manipulation, enable large‐scale parallel culture and drug screening. Coculture systems, involving interactions with vascular, immune, and stromal cells, facilitate in‐depth studies of multicellular and multitissue dynamics. Additionally, the integration of biosensing and bioimaging technologies, such as electrochemical sensors and high‐resolution imaging systems, allows real‐time dynamic monitoring and precise analysis of organoids. These advances provide powerful technical support for drug development, disease modeling, and personalized medicine.^222^

By integrating organoids with organ‐on‐a‐chip technology, researchers can create more accurate human biological models, such as colon and duodenum chips, to fully utilize the potential of organoids. In these organ chips, organoids are typically dissociated into small fragments and seeded onto a porous membrane coated with a specific ECM. Microvascular ECs (MECs) are seeded on the opposite side of the porous membrane. A microfluidic system facilitates the flow of different culture media through each channel to promote cell differentiation, while adjustments in amplitude and frequency simulate intestinal peristalsis. After a few days, epithelial cells form a confluent monolayer in the top channel, and ECs form a complete vascular system in the bottom channel. This method not only improves the cellular morphology and function of organoids, bringing their gene expression closer to in vivo conditions, but also provides a powerful platform to study more complex human biological mechanisms, such as peristalsis, tumor cell migration, and immune cell interactions—challenges that traditional models struggle to address. Combining these technologies enables more precise simulation of the physical and biochemical functions of human organs and opens new possibilities for complex research designs, such as drug permeability testing, studies on colonic inflammation, immune cell recruitment, and CRC cell invasion.^223^, ^224^

The gut‐on‐a‐chip can contain multiple isolated compartments to simultaneously culture IECs and specific microbial strains. This coculture system simulates the interactions between gut microbiota and host cells. For instance, beneficial strains can be cultured in one compartment, while pathogenic strains are cultured in another, to investigate their impacts on gut health. Additionally, by employing precise injection pumps and microfluidic channels, researchers can quantitatively introduce specific amounts of microorganisms and control their distribution and concentration within the chip, thus simulating the colonization and dynamic changes of microbes in the gut.^11^ Organs‐on‐a‐chip technology used to simulate gut microbiota‐related diseases, such as IBD and CRC, has played an important role in developing gut–organ axis chips, particularly for further validation of selected microbes. Researchers constructed an intestinal organ chip model made from polydimethylsiloxane (PDMS) to simulate the physiological environment of the human gut, allowing in‐depth exploration of mechanical forces and microenvironmental factors affecting *Shigella* infection. This organ chip contains a central channel with a stretchable porous membrane and hollow side vacuum channels to mimic mechanical movements like peristalsis. The surface is coated with ECM to support the growth and differentiation of human colonic epithelial cells. Introducing *Shigella* into this realistic 3D intestinal microenvironment revealed that mechanical forces and the 3D structure of the gut significantly enhance bacterial adhesion and invasion, particularly in the simulated intestinal crypts, successfully replicating the pathology of *Shigella*‐induced severe diarrhea.^225^ In inflammation models, PDMS‐based immune‐responsive intestinal chips were used to study the impact of symbiotic gut microbes, such as *L. rhamnosus* and *Bifidobacterium longum*, on gut barrier function and matrix remodeling. This model successfully mimicked the complex microenvironment of the human gut, including oxygen gradients, mucosal immune responses, and microbial vertical distribution. It was observed that during LPS‐induced inflammation, the microbiota exhibited geographical distribution changes, with *B. longum* playing a key role in the anti‐inflammatory process. Additionally, the microbiota indirectly participated in matrix remodeling by influencing collagen fiber orientation and ECM remodeling.^226^ For studying anaerobic bacteria, researchers developed a method using human organ‐on‐a‐chip technology to coculture a complex living human gut microbiota, including obligate anaerobes that require strict anaerobic conditions (<0.5–1% O_2_) to survive, in direct contact with human IECs and their mucus layer. While the primary goal was not to simulate specific regions of the gastrointestinal system, the study proposed that the chip could enable cell cultures from different gut regions (such as the duodenum, jejunum, ileum, and colon) using oxygen concentrations appropriate for each region, potentially allowing the introduction of microbiota specific to those areas. By integrating primary epithelial cells from gut biopsies or patient‐derived iPSCs with the microbiota from the same patient, the future development of patient‐, disease‐, and location‐specific host–microbiota coculture models could promote personalized medicine.^227^

The combination of gut organ chips with other organ chips, such as liver chips, has led to the creation of gut–liver axis organ chips, which simulate interactions between multiple organs in the human body using microfluidic technology to recreate complex physiological and pathological processes in vitro. Microfluidic systems utilize laminar flow principles to maintain stable fluid movement in microchannels, preventing turbulence—similar to how blood flows in microvasculature. By precisely controlling the flow rate and fluid dynamics, researchers can simulate the transport of nutrients and metabolites in the body.^228^ These multiorgan chip systems are connected through intricately designed microchannels and micropumps, allowing the flow of blood and metabolites between different organ models, thereby recreating the complex physiological and pathological interactions between the gut and other organs. The system is also integrated with biosensors to monitor key physiological parameters, such as oxygen concentration, pH, and metabolite levels in real‐time, ensuring the accurate replication of the in vivo environment. This integration allows researchers to effectively study drug absorption, distribution, metabolism, and excretion (ADME) across different organs, as well as investigate disease models involving cross‐organ interactions.^12^, ^229^, ^230^

### 6.1 Microbiome–gut–liver axis chip

The bidirectional communication system between the gut and liver plays a crucial role in maintaining metabolic, immune, and inflammatory functions in the body. Through the portal vein, nutrients, microbial antigens, metabolites (such as SCFAs), and BAs from the gut can be directly transported to the liver. The liver not only processes these gut‐derived substances but also responds by secreting bile and regulating immune responses, which in turn influence the composition and function of the gut microbiota. When the gut barrier is compromised, as in the case of “leaky gut,” harmful substances from the gut can more easily enter the liver, leading to inflammation and disease progression.^231^ Gut microbial metabolites play a key regulatory role in liver pathology, and different metabolites are closely associated with various liver diseases. Short‐chain SCFAs, such as acetate, propionate, and butyrate, generally have positive effects on liver function by improving immune responses and metabolic functions. However, high concentrations of SCFAs may also be linked to the development of hepatocellular carcinoma. The composition of BAs is influenced by the gut microbiota, and an increase in secondary BAs has been associated with the progression of NAFLD and liver fibrosis. TMAO, a metabolite produced by gut bacteria, has been found to increase the risk of atherosclerotic CVD and NAFLD. Tryptophan derivatives, such as indole and its metabolites, regulate immune responses and oxidative stress in the liver, potentially playing a role in NAFLD and other metabolic liver diseases.​^232^, ^233^, ^234^

Liver organ‐on‐a‐chip technology, which simulates the anatomical structure and physiological functions of the liver—particularly characteristics like blood flow and oxygen gradients—has been widely applied in the study of drug toxicity and liver diseases. These chips typically use various types of cellular materials, including human primary hepatocytes, HSCs, liver sinusoidal ECs (LSECs), and KCs, to accurately replicate the liver's microenvironment and function. Some models use primary hepatocytes from mice or rats, while others employ human‐iPSCs‐derived hepatocytes to meet different research needs. In these chips, microfluidic technology simulates blood flow in the liver, with microchannel designs mimicking the liver's vascular network, allowing liver cells to grow and function in a dynamic fluid environment. Peristaltic pump systems are often used to continuously circulate culture medium, simulating blood flow from the portal vein and hepatic artery.^215^, ^235^ Liver‐on‐a‐chip technology has been extensively applied to study drug‐induced liver injury, ALD, NAFLD, and other liver conditions. The liver expresses drug‐metabolizing enzymes at high levels, making it susceptible to drug‐induced liver injury. However, under traditional 2D cell culture conditions, hepatocytes exhibit low drug metabolism activity, making it challenging to assess such injuries effectively. In contrast, liver organ chips better maintain the metabolic activity of hepatocytes and provide a dynamic, precise platform for evaluation by integrating biosensors to monitor cytokine levels and oxygen concentration in real‐time. One liver‐chip model incorporates primary hepatocytes, LSECs, KCs, and HSCs from humans, mice, and dogs. Certain drugs, such as bosentan and fialuridine, show significant toxicity in human cells within the chip, but not in rat cells, highlighting the model's potential for evaluating species‐specific drug‐induced liver injury.^236^ In research on ALD, a multichamber coculture device containing hepatocytes and HSCs was developed. This model simulates alcohol consumption by introducing ethanol‐containing culture medium, resulting in a significant increase in TGF‐β produced by hepatocytes and HSCs after ethanol treatment, which further induced fibrosis progression.^237^ Another liver organ chip model, which includes hepatocytes, HSCs, LSECs, and KCs, was used to simulate the pathological processes of NASH by introducing a lipotoxic culture medium. This model demonstrated lipid droplet accumulation in hepatocytes and increased expression of fibrosis markers in HSCs under lipotoxic conditions.^238^

In the gut–liver axis chip model, independent chambers simulating the intestine and liver are connected through microfluidic channels, recreating the natural circulation of blood and other fluids between the gut and liver. To ensure the accuracy of experimental data, researchers commonly use specially treated silicone materials like PDMS, known for its high elasticity, optical transparency, and gas permeability, which allows for long‐term cell culture in enclosed chambers. During the construction of organ chips, microstructures are first designed using CAD software, followed by the use of photolithography or laser technology to create a template. Soft lithography is then used to mold elastic polymers like PDMS, with micromachining techniques generating complex, multilayered chips. Once the chip structure is complete, target cells (such as intestinal or liver cells) are introduced into the chip, and the microfluidic system is integrated to simulate interactions between multiple organs. While PDMS offers excellent optical properties and biocompatibility, it has some drawbacks, including strong adsorption, lengthy preparation processes, and challenges in large‐scale production. To overcome these limitations, researchers have explored alternative materials like polymethyl methacrylate and cyclic olefin copolymer, which possess good optical properties and chemical stability, making them suitable for mass production. When modeling diseases such as NAFLD, the microenvironment of the gut and liver can be reconstructed on the chip to observe the accumulation of fatty acids in intestinal and liver cells and the subsequent cell damage and death processes. This simulation method not only enhances understanding of the gut–liver axis under physiological and pathological conditions but also provides a powerful platform for drug screening and disease treatment.^239^, ^240^, ^241^

**FIGURE 4:**
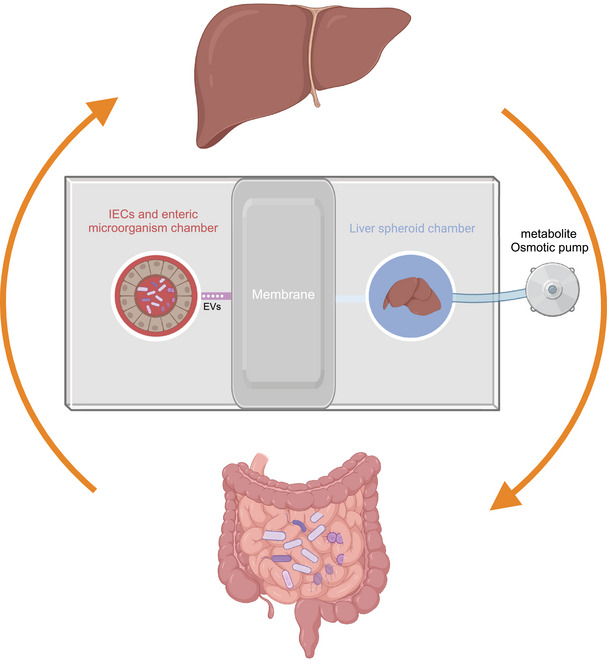
Gut–liver axis chip: simulating interactions between gut microbiota and liver tissue. The gut–liver axis chip mimics in vitro interactions between gut microbiota and liver tissue using a dual‐chamber system. IECs and microorganisms are cultured in one chamber, while HepG2 liver organoids are in the other, separated by a porous membrane for metabolite exchange. Fluid dynamics ensure uniform distribution, supporting long‐term coculture. Microbiota‐derived metabolites, especially from HY7207 probiotics, enhance liver functions such as albumin and urea secretion, with EVs further boosting liver activity without toxicity.

The homeostatic relationship between the gut, gut microbiota, and liver plays a critical role in drug metabolism. Although animal models are widely used in drug toxicity assessments, significant differences between humans and rodents in drug‐metabolizing enzymes and microbial communities within the gut and liver limit their applicability. By utilizing patient‐derived human iPSCs and patient‐specific gut microbiota, organ‐on‐a‐chip platforms can offer personalized predictions of drug metabolism and its effects on patients. First, cells are extracted from a patient's tissue and reprogrammed into stem cells using iPSC technology. These stem cells are then differentiated into specific organ cell types, such as IECs, hepatocytes, and KCs, to build a patient‐specific gut and liver model. Concurrently, stool samples are collected from the patient to culture and analyze the gut microbiota, which is then integrated into the gut model of the organ chip platform to simulate the patient's unique microbial environment. By combining patient‐derived gut and liver cells with their specific gut microbiota, an in vitro model of the gut–liver axis can be constructed on the organ chip platform. This model allows for precise control of cell culture parameters, replicating physiological conditions within the human body. The test drug is introduced into the organ chip platform to observe its metabolic processes within the patient‐specific gut and liver model. By measuring the ADME of the drug, the platform assesses its metabolites, toxicity, and efficacy. High‐throughput screening and computational analysis tools are then used to analyze the platform's data. By comparing the metabolic pathways and results across different patient models, researchers can predict the drug's metabolic characteristics and potential toxicity in individual patients, helping to optimize dosage and treatment plans while avoiding adverse reactions in personalized medicine.^242^ For example, researchers developed a gut–liver axis chip capable of simulating the complex interactions between the gut microbiota and liver tissue in vitro, overcoming the limitations of traditional single‐cell culture systems. This chip uses a dual‐chamber design, with IECs cultured on one side and uniformly sized HepG2 liver organoids on the other, separated by a porous membrane that allows metabolites to pass through. Computational fluid dynamics simulated fluid flow, demonstrating uniform distribution within both chambers, which is crucial for maintaining physiological conditions. The system enabled long‐term coculture of gut microbiota and liver organoids. The experiment showed that when HepG2 organoids were exposed to microbiota‐derived metabolites, particularly those from *HY7207* probiotics, albumin and urea secretion significantly increased, outperforming the WT strain. Under flow conditions, the survival and functionality of HepG2 organoids were maintained, and extracellular vesicles (EVs) derived from the microbiota further enhanced liver function without causing cytotoxicity, even at high concentrations (Figure 4).^243^

### 6.2 Microbiome–gut–brain axis chip

The bidirectional communication between the microbiota, gut, and brain involves neural signals from the vagus and spinal afferent neurons, microbial factors, gut hormones, and cytokines released by the immune system. This communication occurs through multiple pathways, including the autonomic nervous system, enteric nervous system (ENS), hypothalamic–pituitary–adrenal axis, neuroimmune system, and the transfer of metabolites from the gut mucosa into the bloodstream.^244^ The role of gut microbial metabolites in brain pathology has gained increasing attention. SCFAs, such as butyrate, propionate, and acetate, are produced by gut bacteria through carbohydrate fermentation. These metabolites not only promote gastrointestinal function by stimulating the sympathetic nervous system but also regulate neurotransmission by promoting serotonin release from the mucosa. Moreover, SCFAs show potential neuroprotective effects by interfering with the formation of soluble Aβ aggregates in AD. Certain prebiotic fibers like inulin and fructooligosaccharides can promote the growth of butyrate‐producing bacteria, and the butyrate they produce can cross the BBB, protecting neurons and helping to alleviate cognitive impairment and depression symptoms. Gut microbes can also stimulate enteroendocrine cells to produce neuropeptides like PYY, NPY, and CCK, which influence brain function via the bloodstream and directly impact the ENS, affecting neurotransmission and neuroinflammation. Additionally, some gut bacteria produce brain‐active compounds such as folate, serotonin, dopamine, and GABA, which play critical roles in cognition, mood regulation, and neuroinflammation.^245^ Brain‐on‐a‐chip models typically use PDMS as the base material, and biomimetic techniques are employed to construct different regions of the brain. Specifically, brain organoids derived from human PSCs are used to simulate the overall brain structure, while decellularized brain ECM provides a supportive environment. These chip models can incorporate neural stem cells, brain ECs, and pericytes to simulate neuronal networks and the BBB. Additionally, primary human astrocytes and microglia are included to represent brain support cells and immune responses. The combination of these components allows brain chips to precisely model specific brain regions and functions, such as the substantia nigra, brain vasculature, and neuron‐to‐neuron interactions, making them suitable for studying neurodegenerative diseases, stroke, brain infections, and potential therapeutic approaches.^246^

The gut microbiota–brain axis proposes a bidirectional functional relationship between the gut microbiota and the brain. In the study of the microbiota–gut–brain axis (MGBA), organoids have become valuable tools for investigating this complex system. Organoids are 3D self‐organizing structures derived from adult tissue biopsies or iPSCs that accurately mimic key features of human tissues, including in vivo‐like tissue architecture, region‐specificity, and diverse cellular subtypes. Specifically, gastrointestinal organoids contain various functional endocrine cells, especially enterochromaffin cells, which can trigger the ENS and transmit chemosensory signals to the brain.^247^ This enables the gastrointestinal organoid system to replicate the bidirectional communication of the GBA in vitro, making it particularly useful for studying host–microbe interactions, such as those between the host and symbiotic or pathogenic microorganisms. In experimental settings, gastrointestinal organoids often use microinjection techniques to directly deliver microbes or their metabolites to the apical surface of the organoid epithelium. This method preserves the ecological niche required for microbes and is suitable for studying long‐term microbial survival and interaction within organoids.^248^ However, due to the operational complexity and lower reproducibility of microinjection techniques, researchers have developed alternative approaches. For instance, by reversing the polarity of intestinal organoids so that the apical surface is exposed to the culture medium without disrupting the intestinal structure and function, researchers can more effectively study infection patterns of invasive gut pathogens. Additionally, some studies dissociate organoids into monolayers of cells and reseed them onto Transwell inserts or Matrigel/ECM‐coated culture plates to form monolayers with exposed apical surfaces. While these monolayers retain the cellular diversity of organoids, they may not fully capture the 3D microenvironment of in vivo tissues, which limits their suitability for long‐term experiments. Despite their great research potential, organoid models have inherent limitations. These include challenges in replicating age‐related structures and functions of native tissues, batch‐to‐batch size heterogeneity, and the absence of key in vivo components such as vasculature, the ENS, and the immune system. These limitations restrict the application of organoids in studying the microbiota–gut–brain signaling pathway.^249^

The MGBA organ‐on‐a‐chip platform advances these concepts by integrating various cell types from the gastrointestinal system, nervous system, and microbiota to simulate the dynamic interactions between these systems. For instance, guts‐on‐chips have been designed to replicate specific structural and functional features of the intestine, such as the formation of villus‐crypt structures and the production of mucus layers. These chips can continuously supply nutrients and remove waste while integrating vascular and immune system components, as well as gut microbiota, further enhancing their biomimetic capabilities and functionality.^250^ In practical applications, researchers have used patient‐derived iPSCs and organoid‐derived cells to develop advanced models of the gut and BBB/neurovascular unit, better capturing the complexity of intestinal epithelial models in various conditions. Through modular design, gut‐on‐chip platforms can function independently or be fluidically coupled to allow material transfer and functional connectivity between organs, simulating in vivo conditions.^251^

One study developed a MGBA organ‐on‐a‐chip model to simulate brain features associated with AD. This model consists of multiple modules, including a microbiota module, gut module, and brain module. The microbiota module simulates interactions between the microbiota and the host, focusing on how the microbiota influences brain function through the GBA. The gut module replicates the physical and chemical environment of the gut, simulating physiological conditions like fluid shear stress and dynamic epithelial responses. The brain module uses a hydrogel‐based 3D brain cell model, combining collagen with polyethylene glycol or hyaluronic acid to form a semi‐interpenetrating polymer network. This system also incorporates H4 cells overexpressing amyloid precursor protein to replicate AD pathology. The modules are connected through a microfluidic system that allows the transfer of substances and signals across modules. This system is designed to precisely simulate the MGBA in vitro, enabling researchers to study AD's pathogenesis, particularly the potential role of gut microbiota in neurodegenerative diseases.^252^

In another MGBA organ‐on‐a‐chip study, the gut module used human IECs to simulate the intestinal lining. These cells were cultured on a porous PDMS membrane, enabling paracrine communication between cells and further replicating the topological features of the gut surface by introducing microvilli structures. This setup recreated the environment for nutrient absorption, mucus production, and interactions with microbial communities. The ENS module was created by differentiating human ESCs into enteric neurons. These neurons, whose axons penetrated the PDMS membrane, interacted with the gut module. The differentiation process used H9 human ESCs, which were induced into the neuroectoderm pathway through dual SMAD inhibition, followed by WNT signaling activation and retinoic acid exposure to generate neural crest cells. These cells were further differentiated into enteric neurons under the influence of specific growth factors. The enteric neurons were cocultured with IECs to simulate the ANS functions in the gut, coordinating motility and secretion activities. The CNS module cultured CNS neurons and was connected to the ENS module through microchannels. These microchannels guided axonal growth, promoting synaptic connections between CNS and ENS neurons, simulating brain functions, particularly the processing of signals from the gut and ENS modules.^253^

**FIGURE 5:**
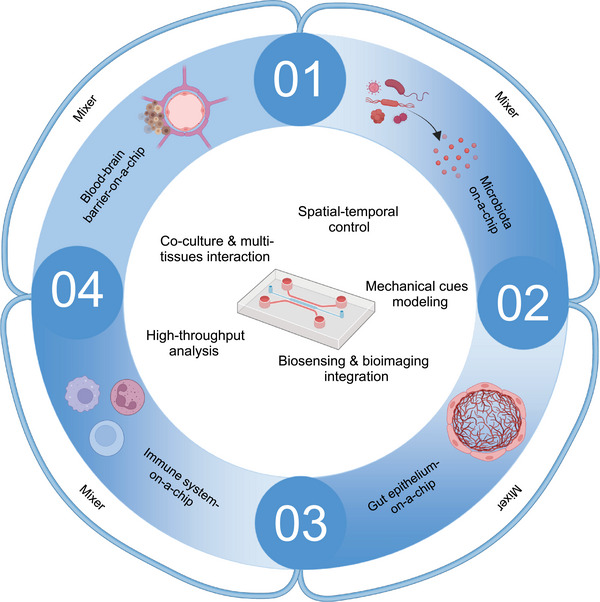
The MINERVA platform: a multiorgan chip for studying GBA. The MINERVA platform is an advanced multiorgan chip system integrating five interconnected organ‐on‐a‐chip devices, including the microbiota, gut epithelium, immune system, blood–brain barrier, and brain. These systems are linked through microfluidic channels, simulating MGBA and its influence on neurodegenerative diseases. The platform models how secretions from gut microbiota traverse the body, impacting the gut, immune system, and BBB, ultimately influencing brain cells such as neurons, astrocytes, and microglia.

The MINERVA platform is an innovative multiorgan chip system that integrates five miniaturized, sensorized, and optically accessible organ chip devices: the microbiota, gut, immune system, BBB, and brain. These devices are connected through microfluidic channels, simulating the MGBA and its role in neurodegenerative diseases. The platform models the transmission pathways of gut microbiota secretions within the body to study how they influence the gut epithelium, immune system, and BBB, eventually affecting neurons, astrocytes, and microglia in the brain. MINERVA offers a unique tool for exploring how gut microbiota secretions impact brain function and contribute to or exacerbate neurodegeneration. Additionally, it provides a more reliable in vitro model for drug screening (Figure 5).^254^

EVs play a key role in the GBA, and organ‐on‐a‐chip technology offers significant potential for studying this bidirectional communication system. EVs in the gut microenvironment, including those from gut cells, bacteria (such as outer membrane vesicles, OMVs, and cytoplasmic membrane vesicles, CMVs), and other microbes, may have a substantial impact on brain physiology and behavior. By simulating the dynamic interactions between the gut and brain, this technology provides a powerful platform for understanding EV‐mediated communication in the GBA. The chips typically consist of IECs and brain MECs. The IECs, as the main component of the gut barrier, are responsible for nutrient absorption and preventing harmful substances from entering the body, while brain MECs form the BBB, regulating the exchange of substances between the bloodstream and brain tissue. These cells are connected through microfluidic channels, allowing researchers to study how gut microbial metabolites pass through the BBB and influence brain function in an in vitro environment. Additionally, the chip design controls fluid dynamics and cell signaling, offering a robust platform for studying interactions between the gut microbiota and the nervous system.^255^

### 6.3 Microbiota–gut–organ axis organ chip and cell death

Using the microbiota–gut–liver axis chip as an example, sampling of drug metabolites can be conducted at the outlet of the gut module and the inlet and outlet of the liver module, which include preliminary metabolites from drugs and gut microbiota, metabolites entering the liver, and those further processed by the liver. High‐performance liquid chromatography (HPLC) is used to separate and quantify drugs and their metabolites, while mass spectrometry (MS) combined with HPLC is used to determine the molecular weights and structures of metabolites.^229^ For studying iron ferroptosis, Sampling of gut microbiota metabolites can be done at the outlet of the gut module and the inlet of the liver module, collecting metabolites produced by gut microbes, such as SCFAs, BAs, and other small molecules, as well as microbial metabolites entering the liver through the portal vein. Gas chromatography‐MS(GC–MS) is used to analyze volatile and semi‐volatile metabolites such as SCFAs, while liquid chromatography‐MS (LC–MS) is used to analyze nonvolatile metabolites such as BAs.^256^, ^257^ Mechanistic studies of ferroptosis involve detecting ferroptosis‐related metabolites and biomarkers in hepatocytes at the outlet of the liver module and detecting intracellular iron levels and oxidative stress markers through cell lysates. Iron ion concentration is detected using colorimetry or atomic absorption spectroscopy, lipid peroxidation is measured using MDA and 4‐HNE, and the redox state is assessed by measuring the GSH to oxidized GSH and levels of ROS.^258^, ^259^ Monitoring the redox state in the gut using wireless sensors allows for the assessment of the reductive or oxidative trends of the chemical environment, thus aiding in understanding the effects of drugs or metabolites on ferroptosis.^260^ Researchers have developed a flow‐through pH sensor to monitor pH changes in liver and heart chips to assess drug toxicity.^261^ Through microfluidic systems, sensors are integrated into the chip, enabling continuous monitoring of the microenvironment. In dynamic monitoring and data analysis, real‐time sensors, such as redox sensors and pH sensors, are used to monitor changes in the redox state and pH within the chip, especially in the gut module.^262^ Multivariate statistical analysis is used to analyze changes in metabolites and biomarkers under different conditions. Mechanical models, combined with experimental data, use mathematical models to simulate the transmission and action mechanisms of drugs and metabolites in multiorgan systems. For example, using multiorgan chip systems to study the interactions of drugs between different organs, the metabolism of the drug CPT‐11 was evaluated in the liver and lung modules using a PK–PD model to assess the metabolic processes and drug effects.^263^ Additionally, by integrating dynamic microfluidic connection systems and multiorgan modules, researchers can achieve precise cross‐organ signal transduction and metabolite transfer, thereby better simulating the complex physiological environment of the human body.^264^

Overall, the microbiota–gut–liver organ‐on‐a‐chip platform (and other microbiota–gut–organ chip systems) provides an effective means for studying the impact of gut microbiota and its metabolites on liver cell death. First, IECs and hepatocytes derived from patient sources or human‐iPSCs are used to build a three‐chamber chip system that simulates the physiological flow of the gut–liver axis. Depending on the research needs, the gut microbiota, drugs, and specific metabolites to be studied are introduced into the gut module for coculture, allowing the distinction between the effects of gut microbial metabolites and the drugs themselves. During operation, the flow rate, pressure, and direction of microfluidic channels must be precisely controlled to simulate the physiological conditions of portal venous blood flow, enabling the transfer of metabolites from the gut module to the liver module. Sensors are used to monitor pH and oxygen concentration in real time, recreating an in vivo‐like cell death environment. To study the effects of gut metabolites on liver cell death, metabolite samples are first collected at the outlet of the gut module and the inlet of the liver module, and volatile and nonvolatile metabolites are analyzed using GC–MS and LC–MS. Next, the liver cell death process is induced in the liver module. For example, liver organoids constructed using iPSCs and gene editing technologies were used to investigate the pathological mechanisms of mitochondrial DNA depletion syndrome in the liver and brain. Research found that liver failure caused by mutations in the deoxyguanosine kinase (DGUOK) gene is closely related to ferroptosis triggered by iron overload. When iron overload was induced using ferric ammonium citrate, cells were found to be highly sensitive to ferroptosis, and ferroptosis inhibitors such as deferoxamine and Fer‐1 were able to reverse this process, demonstrating that liver failure was driven by ferroptosis induced by iron overload.^265^ Finally, biochemical assays and multiomics approaches are employed to detect cell death‐related markers and evaluate the role of gut microbial metabolites in liver cell death.

## 7 TECHNOLOGICAL ADVANCES IN REVEALING GUT MICROBIOTA'S IMPACT

In the analysis of gut microbiota, 16S ribosomal RNA (16S rRNA) sequencing, metagenomics, and metatranscriptomics are commonly used methods. 16S rRNA sequencing is a widely used technique for determining the taxonomic composition of bacterial communities by amplifying and sequencing the 16S rRNA gene. The method uses conserved regions of the 16S rRNA gene as primer targets to detect the presence of different bacteria. However, due to the conserved nature of the 16S rRNA gene among different species, this method has limitations in distinguishing closely related species or strains. Moreover, 16S rRNA sequencing primarily provides taxonomic information and cannot directly offer functional insights, requiring computational prediction to infer functions, which limits the precise evaluation of microbial roles. Additionally, the need for PCR amplification introduces the risk of sequence errors and biases, potentially affecting the accuracy of taxonomic results.​^266^ Metagenomics is a method that involves sequencing all DNA in a sample to comprehensively reveal the microbial community. This technique captures the entire genomic DNA of microorganisms in environmental samples through high‐throughput sequencing, providing complete genetic information, including nonbacterial members. Metagenomics offers species‐level taxonomic resolution and reveals the genetic potential of microbial communities. However, it requires a high sequencing depth, making it costly. Functional redundancy is also a challenge in metagenomics, as different microorganisms may encode the same metabolic functions, making it difficult to link specific functions to specific microbes. Although metagenomics can uncover strain‐level genetic variation, it may not fully capture expression differences between strains.^267^ Metatranscriptomics measures active gene expression in microbial communities by analyzing RNA, particularly mRNA, offering a snapshot of microbial activity and real‐time function. The method works by sequencing mRNA to capture the transcriptional activity of the microbial population, revealing which genes are actively expressed at a specific time point. However, because metatranscriptomics captures dynamic processes, it only provides a brief snapshot of activity, potentially missing a comprehensive view of the microbial community's functional potential. Capturing low‐abundance transcripts requires sufficient sequencing depth, making it an expensive approach. Additionally, the functions of the same species can vary across individuals or environments, complicating the interpretation of results.^268^​

To overcome the limitations of traditional methods, single‐cell RNA sequencing (scRNA‐seq) is gradually being applied to microbiome research. This approach allows for transcriptomic analysis at the single‐cell level, revealing the functional states and gene expression patterns of individual microbes. For example, scRNA‐seq has made significant progress in uncovering microbial diversity and function. Early single‐cell techniques like Drop‐seq and Smart‐seq2 achieved some success in capturing single microbial cells and their transcriptomes, but challenges remain in handling complex microbial communities, particularly due to the higher levels of impurities in microbial samples compared with eukaryotic cell samples.^269^ Drop‐seq and Smart‐seq2, for instance, struggle with high background noise and low cell capture efficiency.^270^ More recent single‐cell transcriptomic technologies, including microSPLiT, PETRI‐seq, MATQ‐seq, and BacDrop, have combined barcoding, plate culture, high‐throughput sequencing, and droplet‐based sequencing to study bacterial transcriptional responses and heterogeneity.^271^, ^272^ For instance, Kuchina et al.^273^ used microSPLiT to analyze the transcriptional heterogeneity of *Bacillus subtilis* across different growth stages. The development of smRandom‐seq has further improved the efficiency of capturing single‐cell RNA from complex microbial communities by optimizing random primer design and microfluidic platforms. smRandom‐seq enables precise analysis of microbial transcriptional activity at the single‐cell level, allowing researchers to understand the role of specific bacteria in processes such as cell death. This technology not only enhances capture efficiency but also reduces cross‐contamination during experiments, thus providing higher‐quality transcriptomic data. smRandom‐seq2, a droplet‐based high‐throughput single‐bacterium RNA sequencing technique, uses random primers and droplet barcoding to capture bacterial RNA and synthesize cDNA. Since prokaryotic mRNA typically lacks poly(A) tails, random primers or specialized rRNA removal strategies are employed to effectively capture bacterial mRNA. During this process, Cas9 is used to remove rRNA, which constitutes the majority of bacterial total RNA, and efficient barcoding and PCR amplification ensure the completion of single‐bacterium transcriptome sequencing within droplets. This method allows for the analysis of gene expression at the single‐cell level in different bacteria, capturing the transcriptional state of individual gut microbes and revealing gene expression patterns under various environmental conditions. This can lead to the clustering of gut microbiota subtypes and functional analysis. For example, smRandom‐seq successfully captured transcriptomic changes in thousands of individual *E. coli* cells, identifying antibiotic‐resistant subpopulations that exhibited differences in SOS response and metabolic pathway gene expression under antibiotic stress. smRandom‐seq can also identify multiple microbial species in human samples. By analyzing bacterial and bacteriophage data from four gut samples, researchers constructed a single‐cell transcriptomic atlas of the gut microbiome, covering 29,742 single cells and 329 species. The study revealed distinct adaptive responses in species from the *Prevotella* and *Roseburia* genera, as well as heterogeneity in the adaptive strategies of *Phascolarctobacterium succinatutens*.^274^, ^275^, ^276^ In summary, 16S rRNA sequencing is used to identify the species present in a microbial community, answering the question of “what species are there.” Metagenomics analyzes the genetic composition of microbial communities, revealing their functional potential and answering “what can the microbial community do.” Metatranscriptomics detects the active gene expression of microbial communities, showing what functions the microbes are currently performing, answering “what is the microbial community doing.” Single‐bacterium transcriptomics, however, delves deeper into the functional heterogeneity at the single‐cell level, revealing “what each individual bacterium is doing,” and highlighting the functional differences between cells.

When identifying target gut microbiota, it is essential to determine their specific metabolic pathways and the metabolites they produce based on functional characteristics and transcriptomic data. This process aids in understanding the role of these microbiota in host metabolism and in identifying key metabolites that may impact health or disease states. However, collecting, processing, and harmonizing such data from multiple sources is a daunting and challenging task. The GMMAD database offers a comprehensive resource that links human diseases, gut microbiota, and gut microbial metabolites, facilitating the prediction and analysis of associations between diseases, microbes, and metabolites.^277^ Researchers often use linear models and random effects models to control for differences between datasets and to validate metabolite associations across multiple studies. First, a linear model is applied to estimate the association between the abundance of a specific genus and metabolite levels. In this model, metabolite levels are treated as the dependent variable, and genus abundance is the independent variable, while controlling for other factors such as study group differences that might influence the results. To handle non‐normal data distributions, metabolite data are usually log‐transformed, and genus abundance data are transformed using an arcsine square root transformation. This allows researchers to independently test the association between genus and metabolite within each dataset. Next, the authors use a random effects model to combine results from multiple datasets. By using partial correlations from each dataset as effect sizes, the random effects model (REM) can account for variability between studies, providing a more robust evaluation of the global association between genus and metabolite. If the model's FDR‐corrected *p*‐value is below 0.1 and the effect sizes are consistent in direction, the genus–metabolite pair is considered to have a significant and consistent association. This approach enables researchers to identify metabolites that show significant associations across multiple studies, increasing the credibility of these associations and uncovering broadly applicable microbe–metabolite links.^278^

**FIGURE 6:**
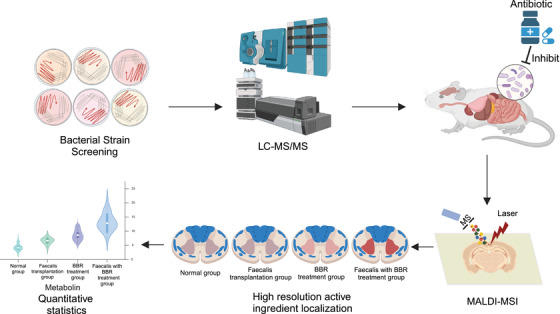
Workflow for investigating the role of gut bacteria in dopamine production using MALDI–MS imaging.

To further determine the spatial distribution of metabolites and uncover the role of the gut–organ axis, spatial metabolomics aims to reveal the spatial distribution of metabolites within tissues, enhancing our understanding of the spatiotemporal characteristics of biological processes. This field combines metabolomics with imaging technologies, primarily using high‐resolution tools such as mass spectrometry imaging (MSI). These techniques allow researchers to precisely map the distribution of metabolites within tissue sections and link these patterns to tissue structure and function.^279^ Spatial metabolomics encompasses various types based on different MS techniques, including matrix‐assisted laser desorption/ionization MS (MALDI–MS), secondary ion MS (SIMS), and time‐of‐flight secondary ion MS (ToF–SIMS). These methods provide detailed images of metabolite distribution at the subcellular level. MALDI–MS is the most commonly used technique, suitable for detecting a wide range of metabolites, while SIMS excels in providing high spatial resolution images. ToF–SIMS allows imaging at the nanoscale. In addition to these standard techniques, nanoelectrospray ionization MS (NanoSIMS) offers further improved spatial resolution, making it particularly useful for analyzing metabolites in single cells or microstructures. Other techniques, such as desorption electrospray ionization MS (DESI–MS) and laser ablation electrospray ionization MS (LAESI–MS), are matrix‐free and are used to analyze the distribution of metabolites in biological tissues, providing high sensitivity without the need for labeling. Airflow‐assisted desorption electrospray ionization MSI (AFADESI–MSI) is another innovative technology that operates under ambient conditions, allowing for in situ analysis of biological tissues without requiring a high‐vacuum environment. AFADESI‐MSI is particularly advantageous for studying the spatial distribution of components in Traditional Chinese Medicines within tissues.^280^ For example, a study utilized AFADESI–MSI to investigate the pharmacological effects and mechanisms of the ginseng‐Schisandra herbal pair in an AD mouse model. Spatial metabolomics analysis of brain tissue sections revealed 28 AD‐related biomarkers, including those associated with neuroinflammation and energy metabolism disorders. The study highlighted the roles of small molecules such as ginsenosides and lignans in the CNS, underscoring their potential therapeutic effects in the context of AD.^281^ Recent research highlights that the gut microbiota serves as a novel source of dopamine precursors in the body. Specifically, the study demonstrated that oral administration of BBR significantly increased the production of dopa and dopamine by gut bacteria in mice. This increased production was found to be dose and time dependent, with dopa produced in the gut entering the bloodstream and subsequently being converted into dopamine in the brain. The study utilized spatial metabolomics and LC–MS nontargeted metabolomics to reveal that BBR's effect on dopamine levels was mediated through gut microbiota. Further experiments confirmed that the gut bacteria, particularly *Enterococcus faecalis* and *Enterococcus faecium*, were critical for this process, as antibiotic treatment that reduced gut bacteria also diminished BBR's effects. Moreover, clinical studies on hyperlipidemia patients corroborated these findings, showing that BBR treatment increased blood dopamine levels, likely through its modulation of gut microbiota composition (Figure 6).^282^

Gut microbiota not only influence diseases through their metabolites but also play a role via their migration. Studies have shown that spatial metabolomics can map metabolic heterogeneity within lung tumors. Combined with 16S rDNA sequencing, this approach explored the anticancer mechanism of *A. muciniphila*. Blood samples collected at different time points revealed that *A. muciniphila* migrates from the gut to cancer tissues through systemic circulation.^283^ Integrating single‐cell transcriptomics with spatial transcriptomics and organ‐on‐a‐chip technologies offers significant advantages for research. By placing tissue sections containing capture probes on slides for transcriptome sequencing, these probes bind to mRNA and record its location within the tissue, generating a spatial map of gene expression at single‐cell resolution. This method allows researchers to uncover the distribution and interactions of different microbial types and states within tissues.^284^, ^285^

## 8 CONCLUSIONS AND PROSPECTS

In gut microbiota research, studies often begin by examining the effects of environmental and external factors on the host. These effects may pertain to specific pathological conditions or normal physiological states. To investigate how environmental factors influence the host or to further explore the metabolic processes impacted by changes in gut microbiota abundance, especially regarding how specific microbes exert influence, researchers must move beyond reliance on antibiotics or GF mouse models to draw conclusions. While these methods have revealed the broad role of gut microbiota, they have significant limitations. The nonspecific effects of antibiotics lead to reduced microbial diversity, potentially masking the function of specific microbes and making it difficult to discern their roles in specific physiological or pathological states. Additionally, antibiotics may directly affect the host system, such as by influencing immune responses or gut barrier function, further complicating the dependence on microbiota.^286^, ^287^

GF mouse models, while providing a host environment free from microbial interference, exhibit significant differences in immune and metabolic systems compared with normal hosts, making it difficult to accurately reflect microbiota functions under natural conditions. For instance, studies have shown that GF mice have different metabolic responses to high‐fat diets compared with conventional mice, largely due to the absence of specific bacteria that regulate energy metabolism, such as those producing SCFAs. Therefore, these traditional methods cannot effectively resolve the specific roles of microbes in pathological states.^288^, ^289^

Gut microbiota play a key role in host nutrient absorption, particularly by converting otherwise indigestible substances into absorbable forms. Through their metabolic activities, gut microbiota transform large molecules or precursor substances into smaller or bioactive forms, allowing them to more easily cross the intestinal barrier into the bloodstream. For example, dietary fiber is fermented by gut microbiota to produce SCFAs, which can be directly absorbed by the host.^290^, ^291^ Additionally, gut microbiota can enzymatically convert precursors of phytochemicals or drugs into active forms, conferring biological functions. However, gut microbiota are not the only factor influencing nutrient absorption. The host's own digestive enzymes and transport proteins also play critical roles in breaking down nutrients and facilitating absorption. For instance, pancreatic enzymes assist in protein breakdown in the gut, promoting their absorption.^292^ Furthermore, the functionality and permeability of the gut barrier directly determine whether substances can enter the bloodstream. Tight junction proteins and the structural integrity of the gut barrier are crucial for regulating permeability, and certain diets or medications can alter permeability, thus affecting absorption.^293^

The application of isotope labeling in gut microbiome research has become a valuable tool for investigating microbial functions and metabolic pathways. For instance, stable isotope probing uses labeled carbon or nitrogen to trace microbial metabolic activities, revealing how microbes metabolize specific substrates. However, this technique has limitations, including its complex and costly experimental design, challenges in analyzing labeled spectral data, and the inability of in vitro conditions to fully replicate the complexity of in vivo microbial interactions, which can lead to biased results.^294^, ^295^

Bacterial pure culture and metabolite analysis, through culturing target microbes in vitro and detecting their metabolites using MS, can eliminate environmental factors and directly confirm metabolite sources. However, this in vitro environment may not fully replicate the host's complex gut environment, potentially overlooking interactions between microbes and the host or other microbes. Some metabolites may only be produced through specific host–microbe interactions, which in vitro experiments cannot capture.^296^ Gene editing and functional validation, such as knocking out metabolic pathway genes in microbes and analyzing metabolite production, help verify microbial metabolic roles. Labeled metabolite injection experiments, traced via MS, allow direct observation of microbial metabolism. However, despite advances like CRISPR–Cas9, there are limitations. Many gut microbes, especially anaerobes, are difficult to culture in vitro, restricting gene editing applications. Additionally, the gut's complex environment and microbial–host interactions are hard to replicate in vitro, meaning functional outcomes in the host may differ. Microbial diversity and horizontal gene transfer, via plasmids or transposons, further complicate precise gene editing. Efficient delivery of gene‐editing tools in the gut without harming the host remains challenging.^297^

To address these limitations, microbiota transplantation experiments are often combined with selected microbial strains. While healthy donor microbiota can be transferred to recipient models to study microbial effects, identifying the specific impact of individual strains is challenging within the mixed microbiota. Complex microbial backgrounds may mask or amplify certain microbial functions. Donor health and microbiota composition significantly influence outcomes, with improper donor selection potentially leading to inconsistent results, especially in cross‐species or cross‐individual transplants. Handling and storage (e.g., freezing, thawing) can affect microbial diversity and function, influencing experimental reliability. Variables such as administration methods (oral or rectal) and dosage also affect results. Standardized protocols are still under development, making experimental reproducibility difficult. Even after successful transplantation, donor microbiota colonization in the recipient can vary due to factors like the recipient's gut environment and immune status, leading to uncertain outcomes.^298^, ^299^, ^300^

The microbiota–gut–organ axis chip technology, as an innovative in vitro model, demonstrates significant advantages in studying the effects of gut microbiota on host organ systems. By simulating the gut microenvironment and integrating multiple organ modules, this chip can accurately replicate the metabolic processes of microbiota in different organs and their interactions with the host. Its primary advantage lies in the precise control of experimental conditions, which eliminates the complex variables of in vivo models and allows for the direct analysis of specific microbial impacts on target organs. Additionally, the microbiota–gut–organ axis chip enables high‐throughput screening, greatly improving experimental efficiency.^301^, ^302^

While in vitro models offer controlled environments for studying microbiota interactions, they cannot fully replicate the complexity of a host's physiology, making parallel validation with in vivo models essential. Microbial transplantation or labeled metabolite tracking in GF animals validates in vitro findings, with in vivo experiments highlighting microbiota roles in dynamic physiological states. Combining both approaches mitigates individual limitations, providing a more complete understanding. Additionally, spatial omics technologies, such as spatial metabolomics, allow for precise localization of microbial–host interactions by identifying where metabolites are produced in different gut or organ regions. This spatial insight enhances the understanding of microbiota effects on host health, especially in disease contexts. Future research should integrate multiomics data—such as transcriptomics, metabolomics, and proteomics—along with spatial omics and single‐bacteria transcriptomics. The use of artificial intelligence and machine learning can extract meaningful biomarkers and pathways from large datasets, advancing personalized treatment strategies for gut microbiota‐related diseases.

## AUTHOR CONTRIBUTIONS

*Conceptualization; investigation; writing—original draft; writing—review and editing*: Yusheng Zhang. *Methodology; data curation*: Hong Wang. *Formal analysis; visualization; methodology*: Yiwei Sang. *Validation*: Mei Liu. *Resources*: Qing Wang. *Conceptualization; project administration*: Hongjun Yang. *Project administration; supervision; funding acquisition*: Xianyu Li. All authors have read and approved the final manuscript.

## CONFLICT OF INTEREST STATEMENT

The authors declare that they have no known competing financial interests or personal relationships that could have appeared to influence the work reported in this paper.

## ETHICS STATEMENT

Not applicable.

## Data Availability

Not applicable.
